# Positive selection on schizophrenia-associated *ST8SIA2* gene in post-glacial Asia

**DOI:** 10.1371/journal.pone.0200278

**Published:** 2018-07-25

**Authors:** Naoko T. Fujito, Yoko Satta, Masaya Hane, Atsushi Matsui, Kenta Yashima, Ken Kitajima, Chihiro Sato, Naoyuki Takahata, Toshiyuki Hayakawa

**Affiliations:** 1 School of Advanced Sciences, SOKENDAI (The Graduate University for Advanced Studies), Hayama, Kanagawa, Japan; 2 Bioscience and Biotechnology Center, Nagoya University, Aichi, Japan; 3 Primate Research Institute, Kyoto University, Aichi, Japan; 4 Graduate School of Systems Life Sciences, Kyushu University, Fukuoka, Japan; 5 Faculty of Arts and Science, Kyushu University, Fukuoka, Japan; Tokyo Daigaku, JAPAN

## Abstract

A number of loci are associated with highly heritable schizophrenia and the prevalence of this mental illness has had considerable negative fitness effects on human populations. Here we focused on one particular schizophrenia-associated gene that encodes a sialyltransferase (ST8SIA2) and is expressed preferentially in the brain with the level being largely determined by three SNPs in the promoter region. It is suggested that the expression level of the *ST8SIA2* gene is a genetic determinant of schizophrenia risk, and we found that a geographically differentiated non-risk SNP type (CGC-type) has significantly reduced promoter activity. A newly developed method for detecting ongoing positive selection was applied to the *ST8SIA2* genomic region with the identification of an unambiguous sweep signal in a rather restricted region of 18 kb length surrounding the promoter. We also found that while the CGC-type emerged in anatomically modern humans in Africa over 100 thousand years ago, it has increased its frequency in Asia only during the past 20–30 thousand years. These findings support that the positive selection is driven by psychosocial stress due to changing social environments since around the last glacial maximum, and raise a possibility that schizophrenia extensively emerged during the Upper Paleolithic and Neolithic era.

## Introduction

Schizophrenia is a highly heritable mental illness that causes marked social impairment. More than 100 loci are associated with schizophrenia (e.g., [1]), and environmental risk factors also interact with such genetic risk factors toward development of the illness [2]. Schizophrenia affects approximately 1% of the human population worldwide and its onset typically occurs during the period of late adolescence and early adulthood. Thus, schizophrenia is a prevalent mental illness with serious negative fitness effects and has posed an evolutionary paradox in human evolution.

The ST8 alpha-*N*-acetyl-neuraminide alpha-2,8-sialyltransferase 2 (ST8SIA2) gene (15q26.1) encodes a sialyltransferase that synthesizes polysialic acid (PSA) in the brain [3]. Sialic acids are a family of nine-carbon monosaccharides found on the outer end of glycan chains on the cell surface as well as secreted molecules of the deuterostome lineage [4]. In general, sialic acids have important roles as ligands in cell-to-cell communication. PSA is a linear homopolymer of sialic acids that exhibits a degree of polymerization ranging from 8 to 400. PSA shows highly restricted expression in the brain during embryonic and post-neonatal development, and also persists in the distinct regions of adult brain where neural plasticity, remodeling of neural connections, or neural generation is ongoing [3]. The major carrier of PSA is neural cell adhesion molecule (NCAM), and polysialylated NCAM (PSA-NCAM) participates in neurite outgrowth, synapse formation, and plasticity. In addition to the role in regulation of cell-cell communication, it is suggested that PSA is involved in the functional regulation of ion channels and neurologically active molecules [3]. ST8SIA2 thus contributes to a wide variety of neuronal events by producing PSA, and plays an important role in mental activities [3].

**Fig 1:**
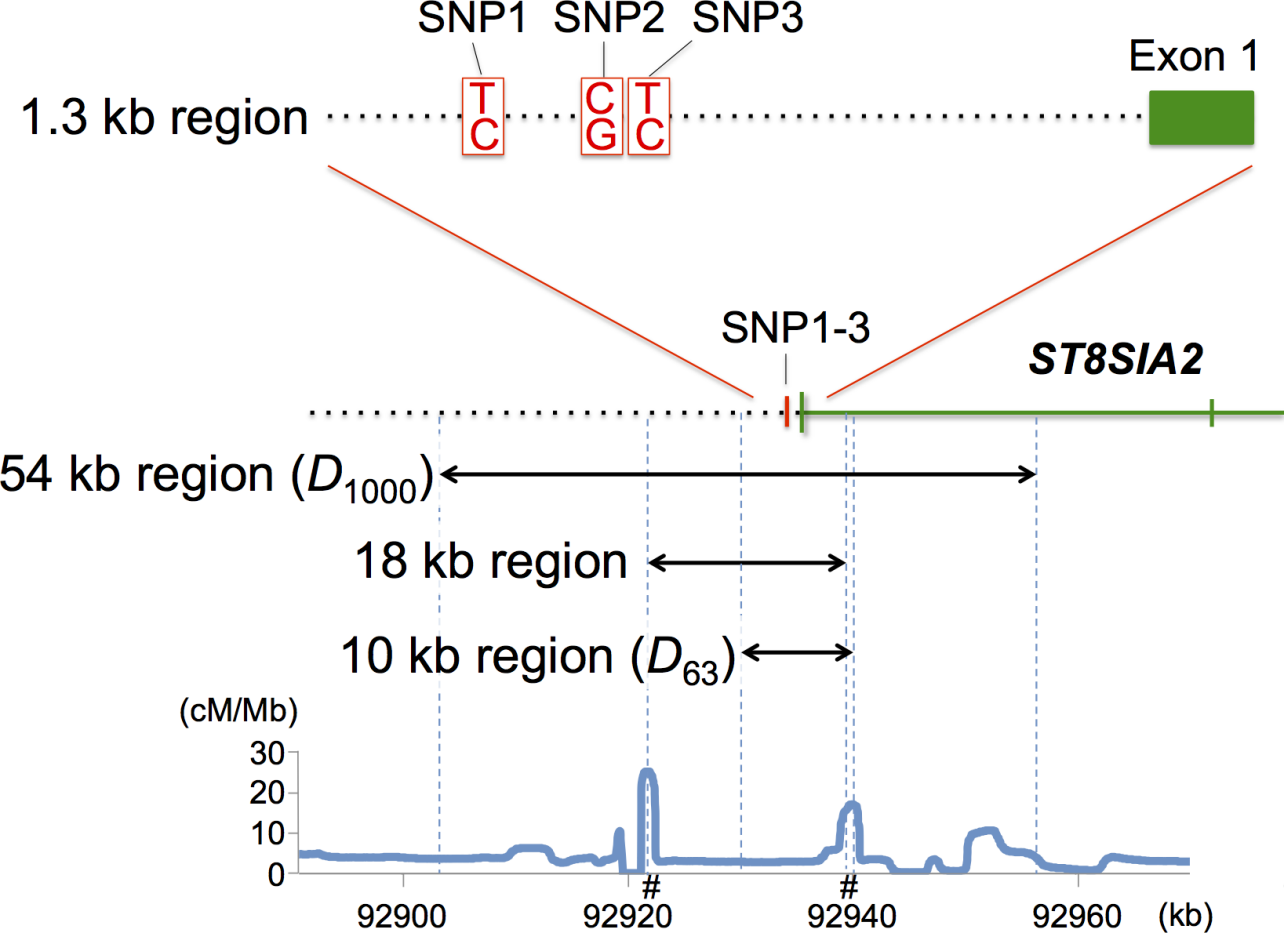
Genomic region surrounding three *ST8SIA2* promoter SNPs. The 1.3-kb region used for the promoter assay and the 10-kb region in *D*_63_ are depicted in the two upper panels. The 54-kb and 18-kb regions in *D*_1000_ (double-headed black arrows) are also depicted in the bottom panel, together with estimated mean recombination rates (cM/Mb). The 18-kb region is defined as the region between two recombination hotspots flanking the three promoter SNPs. A # mark on the X axis represents the location of a recombination hotspot.

Several single-nucleotide polymorphisms (SNPs) at the *ST8SIA2* locus are associated with multiple mental diseases including schizophrenia [2]. Furthermore, *ST8SIA2* gene deficiency in mice results in decreased social motivation and increased aggressive behavior (i.e., schizophrenia-like phenotypes) [5, 6]. These suggest that ST8SIA2 is a primordial molecule responsible for establishing suitable social behavior. Three SNPs (SNP1 at rs3759916, SNP2 at rs3759915, and SNP3 at rs3759914) in the upstream region of the *ST8SIA2* gene (Fig 1) are associated with schizophrenia risk and involved in promoter activity [7–9]. Only the CGC promoter type (C in SNP1, G in SNP2, and C in SNP3) is consistently identified as a non-risk type for schizophrenia in all populations examined (Japanese, Chinese, and Spanish) in contrast to other promoter types such as TGT-, TCT- and CGT-types that are identified as risk types in at least one population [7–9]. It is also found that the promoter activity of the CGC-type is different from the TGT-type [7]. Since the quantity of PSA-NCAM has been suggested as a factor involved in schizophrenia [10], it is expected that the promoter activity of *ST8SIA2* gene is a genetic determinant of schizophrenia risk through controlling the amount of enzyme that regulates PSA quantity.

To gain insight into an evolutionary basis of schizophrenia prevalence, we focus on the CGC-type (a non-risk type) of the *ST8SIA2* gene because of its functional involvement in the illness. Using a novel statistical method, we examine whether or not the non-risk type has been subjected to positive selection in particular environments. Based on the promoter activity, geographic distribution, and gene tree analyses, we also elucidate the evolutionary history of the CGC-type in the human lineage.

## Materials and methods

### Genomic DNA samples

Sixty-three human genomic DNA samples containing indigenous populations (see S1 Table) were purchased from Coriell Cell Repositories (Camden, NJ, USA). Chimpanzee and gorilla genomic DNA samples were a generous gift from then Professor Jan Klein of the Max-Planck Institute for Biology (Germany).

### Typing of the three promoter SNPs in human populations by direct sequencing

To identify *ST8SIA2* promoter types of all 63 individuals, approximately 4 kb sequences surrounding the three promoter SNPs were amplified by genomic PCR using ExTaq DNA Polymerase (TaKaRa, Otsu, Japan) with a pair of PCR primers (STXF1H and STXR1H; see S2 Table). These primers were designed based on the human genomic sequence from NCBI database (https://www.ncbi.nlm.nih.gov). PCR reactions were performed with 50 pmol of each primer and 1 μl of genomic DNA solution in a total volume of 50 μl containing 200 μM dNTPs and 1 μl PrimeSTAR GXL DNA Polymerase (TaKaRa). PCR conditions were: denaturation at 94°C for 1 min, followed by 40 cycles at 98°C for 10 s and 68°C for 4 min, with a final extension at 72°C for 10 min. After degradation of PCR primers with ExoSAP-IT™ PCR Product Cleanup Reagent (Thermo Fisher Scientific, Waltham, MA, USA), amplified products were directly sequenced using an ABI PRISM 3100 Genetic Analyzer (Applied Biosystems, Foster City, CA, USA) with BigDye Terminator v3.1 Cycle Sequencing Kit (Applied Biosystems). Haplotype sequences including the three promoter SNPs were then determined by sub-cloning.

### Sequencing of the human promoter haplotypes by sub-cloning

To determine haplotype sequences from the same 63 human samples, we examined a genomic region (10,219 bp; see Fig 1) that contains the three promoter SNPs at its center ranging from chromosome position 92930766 to 92940985 on human chromosome 15 (GRCh37). This approximately 10-kb region was amplified by PCR with PrimeSTAR GXL DNA Polymerase under the following conditions: denaturation at 94°C for 1 min, followed by 40 amplification cycles at 98°C for 10 s and 66°C for 10 min, and ending with an extension at 68°C for 10 min. PCR primers (STXF0H-2 and STXR0H-2; see S2 Table) were designed based on the human genomic sequence from NCBI databases. Amplified products were gel-purified by QIAquick Gel Extraction Kit (QIAGEN, Hilden, Germany), and subsequently cloned with the Zero Blunt PCR Cloning Kit (Life Technologies, Carlsbad, CA, USA). Subcloned DNA fragments were extracted with QIAprep Spin Miniprep Kit (QIAGEN) and sequenced in a similar way as mentioned above. More than three clones were sequenced for each haplotype at least twice in both directions. From the 126 chromosomes, we determined haplotypes for 91 chromosomes so that at least a single chromosome from each sample was obtained.

### Typing of the three SNPs of the promoter region in chimpanzees and gorillas

To identify the *ST8SIA2* promoter types of six chimpanzees and 14 gorillas, an approximately 800 bp fragment of the promoter region was obtained with genomic PCR. PCR primers (STXF1 and STXR2; see S2 Table) were designed based on chimpanzee and gorilla genomic sequences from NCBI databases. PCR reactions were performed with 20 pmol of each primer and 1 μl of genomic DNA solution in a total volume of 50 μl, including 200 μM dNTPs and 0.5 μl ExTaq DNA polymerase in PCR buffer containing 2 mM MgCl_2_. PCR conditions were: denaturation at 95°C for 5 min, followed by 40 cycles at 95°C for 1 min, 66°C for 1 min, and 72°C for 1 min, and then a final extension at 72°C for 10 min. Amplified products were directly sequenced in a similar way as mentioned above.

### The three promoter SNPs in other nonhuman primates

The three promoter SNPs were examined in the bonobo (panpan 1.1; Release 102), baboon (Panu_3.0; Release 103), rhesus monkey (Mmul_8.0.1; Release 102), and green monkey (Chlorocebus_sabeus 1.1; Release 100) using genome sequences available in the NCBI database.

### *ST8SIA2* promoter activity

The three promoter SNPs are within a 300-bp stretch in the promoter region of *ST8SIA2*, approximately 700 bp upstream of exon 1 (Fig 1). *ST8SIA2* promoter activities in humans and African apes were examined with an *in vitro* luciferase reporter system. Promoter sequence fragments (approx. 1.3 kb) were obtained from human and great apes by PCR. Human homozygous individuals for TCT (NA13617; see S1 Table), CGT (NA13597; see S1 Table), CGC (NA13598; see S1 Table), and TGT (NA19208; see S1 Table) promoter types were chosen as templates. PCR primers (STXF8 and STXR6; S2 Table) were designed based on genomic sequences of humans, chimpanzees, and gorillas from NCBI database. PCR reactions were performed with 20 pmol of each primer and 1 μl of genomic DNA solution in a total volume of 50 μl, including 200 μM dNTPs and 1 μl PrimeSTAR GXL DNA polymerase (TaKaRa) in PCR buffer containing 2 mM MgCl_2_. Genomic PCR conditions were: denaturation at 98°C for 1 min, followed by 40 cycles of 98°C for 10 s, 66°C for 15 s, and 68°C for 3 min, with a final extension at 68°C for 10 min. To introduce restriction sites into amplified fragments, the primer pair of STXproF1E and STXproR1B (S2 Table) was used for a second round of PCR. Second PCR reactions were performed with 20 pmol of each primer and 2 μl of genomic PCR product in a total volume of 50 μl, including 200 μM dNTPs and 1 μl PrimeSTAR GXL DNA polymerase in PCR buffer containing 2 mM MgCl_2_. PCR conditions were: denaturation at 98°C for 1 min, followed by 35 cycles of 98°C for 10 s, 60°C for 15 s, and 68°C for 2 min, and a final extension at 68°C for 10 min. Amplified DNA fragments were digested with *EcoR*I and *BamH*I, and cloned into the vector, pMetLuc2-Reporter plasmid vector (Clontech, Mountain View, CA, USA). A sequence corresponding to the transmembrane domain of the plasmid was successively eliminated by additional PCR. Plasmids were linearized with phosphorylated pmST8SIA2-noTM primer (S2 Table) and phosphorylated MetLuc2-ATG primer (S2 Table) by KOD-Plus-Neo polymerase (TOYOBO, Fukui, Japan). Linearized plasmids were self-ligated and pMetLuc2-ST8SIA2 promoter plasmids were obtained. The reporter plasmid with cloned promoter sequence fragment was co-transfected into IMR-32 (human neuroblastoma) cells with the pSEAP2-Control plasmid (Clontech). The vector, pMetLuc2-Control (Clontech), was used as a positive control, and pMetLuc2-Reporter (lacking promoter sequence) as a negative control.

### DNA sequence data

Two datasets of phased sequences were constructed. DNA sequences of approximately 1 Mb spanning the three promoter SNPs and covering 2,504 individuals (S3 Table) were retrieved from phase 3 of the 1000 Genomes Project database [11]. For the phase 3 sequences, the switch error rate is 0.56%, and the mean inter-switch distance is 1062.1 kb. Since the length of sequences used in this study was at most 200 kb, their sequence quality should be satisfied. Furthermore, the quality score is 100 for all sites used in this study, which means that the accuracy of base calling is over 99.9%. Additionally, 91 haplotype sequences of 10 kb length were determined from 63 worldwide samples (see the section of “Sequences of the human promoter region”). The former dataset is hereafter abbreviated as *D*_1000_, and the latter dataset as *D*_63_. We classified DNA sequences from *D*_1000_ into five meta-populations: Africa (AFR), Europe (EUR), East and Southeast Asia (EAS), South Asia (SAS), and America (AMR) (S3 Table). Sequence analyses first focused on an 18-kb region that is sandwiched between recombination hotspots (see below) in *D*_1000_, and the 10-kb region in *D*_63_ (Fig 1). The 18-kb region was extended in both directions and a 54-kb region (Fig 1) was then subjected to neutrality tests with summary statistics, SFS, and *F*_ST_. DNA sequences of approximately 1 Mb were also analyzed for relative extended haplotype homozygosity (REHH), homozygosity tract length (HTL), and *F*_c_ (see below).

### Recombination rate

Recombination rates were calculated by the LDhat 2.2 program [12] for SNP data from Han Chinese in Beijing, China (CHB), Japanese in Tokyo, Japan (JPT), Utah Residents (CEPH) with Northern and Western European Ancestry (CEU), and Yoruba in Ibadan, Nigeria (YRI) populations of *D*_1000_ (Fig 1 and S1 Fig). The rates of recombination hotspots were estimated as 7–11 cM/Mb (S1 Fig) whereas the background recombination rate was estimated as 1–4 cM/Mb. The recombination hotspots were commonly observed in such non-Africa populations as CHB, JPT and CEU (S1 Fig). Overall, the average recombination rate (3.3 cM/Mb) was used in simulation as a representative in the region under study.

### ADMIXTURE analysis

The ADMIXTURE program [13] was used to estimate the ancestry of the 18-kb *ST8SIA2* promoter region. In the 18-kb region, 546 SNPs were available for the ADMIXTURE analysis.

### Testing neutrality by site frequency spectrum (SFS)

The segregating sites in a sample of size *n* (e.g., 694 for AMR as the smallest *n* among the five meta-populations in *D*_1000_) were binned (e.g., [14]) into eight classes according to the following number (*i*) of derived alleles at each SNP site: *i* = 1, 2 ~ 3, 4 ~ 9, 10 ~ 25, 26 ~ 68, 69 ~ 185, 186 ~ 503, and 504 ~ (*n* − 1). The ancestral allele in each SNP is defined in the dataset of the 1000 Genomes Project, and referred in this study. Under the standard neutral model of constant population size *N*_*e*_, this binning locates nearly the same number of segregating sites in each class, as the expected number (*E*{*ξ*_*i*_}) of segregating sites each exhibiting *i* derived alleles is given by *θ*/*i* where *θ* = 4*N*_*e*_*μ* (e.g., [14, 15]). The neutrality test was performed based on the relative SFS (rSFS) as well as summary statistics. Here rSFS was defined as the ratio of the observed *ξ*_*i*_/*S* ($S=\sum_{i=1}^{n-1}\xi_{i}$) to 1/*ia*_*k*_) where $a_{k}=\sum_{i=1}^{k-1}1/i$.

Simulation was carried out using ms [16] to examine the SFS of neutral mutations under both the standard model of constant size and the demographic model proposed by Schaffner et al. (2005) [17]. In each simulation, a region was assumed to contain a site of our interest and to be in strong linkage disequilibrium (LD) so that the focal (core) site could be located anywhere as long as it satisfied the condition for a specified derived-allele frequency (i.e., 35%). The specified number of segregating sites was placed on a coalescent tree. For instance, as the observed number of segregating sites in EAS is 160 in the 18-kb promoter region, the same number of mutations was randomly placed on a simulated coalescent tree. This allowed us to compare SFSs with the same number of segregating sites even under different demographic models. The command line was “./ms 1008 4000 -s 160” for the standard model of constant size and “./ms 1008 4000 -s 160 -eN 0.001 0.077 -eN 0.004745 0.007 -eN 0.004995 0.077 -eN 0.0084975 0.006 -eN 0.0087475 0.24 -eN 0.0425 0.125” for the demographic model of changing population size.

### Detecting selective sweep

To detect sweep signals, a new method was developed based on both SFS and LD information, the details of which will be presented elsewhere. Briefly, it divides a sample of *n* homologous chromosomes into two mutually exclusive groups defined at a core site. In the case of *ST8SIA2*, the “core sites” are the three promoter SNPs that define the CGC and nonCGC groups. Calculation was done at the *k*-th SNP site for the number (*n*_*k*_) of derived alleles in the whole sample and the number ($n_{k}^{CGC}$) of derived alleles that are associated with the CGC group. The *n*_*k*_ can range from 1 to *n* − 1, and the $n_{k}^{CGC}$ can range from 0 to *n*_*k*_ or the copy number of the CGC-type (whichever is smaller). These numbers were then transformed to “barcodes” that represent SNP information by two-tone colored heights. The barcode representation of *n*_*k*_ and $n_{k}^{CGC}$ differs from SFS or rSFS in that it preserves not only spatial information about SNP sites but also information about LD with the core site. This method also differs from rSFS in that SNP information is stratified in eight layers that are on average in proportion to allele ages. The LD information could be used to define a core region around the core CGC SNP sites.

To quantify variability within the CGC-type group (henceforth intra-allelic variability abbreviated by IAV), a statistic (*F*_*c*_) was defined by

$$F_{c}=\frac{\sum_{k\in c}n_{k}^{CGC}}{\sum_{k\in c}n_{k}}$$ (1)

for $n_{k}^{CGC}$ and *n*_*k*_ at all SNP sites in specified frequency classes (*c*), to the exclusion of not only the own class to which the core site belongs but also the classes higher than the core class. This exclusion is essential because the core frequency class contains mutations that accumulated in a common stem lineage of the CGC group and do not possess any useful information on internal structure of descendant allelic lineages. In the present case, as the CGC-type has a frequency of 0.35 or 349 copies in the EAS meta-population, it belongs to class 7. Thus, the *F*_*c*_ is computed in the classes lower than class 7 and expressed as *F*_<*c*7_. It is also important to note that the *F*_*c*_ is computed in the 18 kb core region of the CGC-type. A small value of *F*_<*c*7_ is a signal for the action of positive selection (S2 Fig). To evaluate the statistical significance of an observed *F*_*c*_ value or a type I error, simulation was carried out under neutrality for the CGC core region without recombination. At least 1,000 replications were performed to obtain the distribution of *F*_<*c*7_. Needless to say, this *F*_*c*_-based method could unambiguously detect signals of selective sweep in such genes (*LCT*, *OCA2*, and *EDAR*; [18]) that have been demonstrated as targets of positive selection [19].

### Relative extended haplotype homozygosity (REHH)

To calculate REHH or the EHH ratio of derived to ancestral alleles [20], SNP3 was used as a single core site, because unlike SNP1 and SNP2, C at SNP3 is perfectly associated with the CGC-type. In a meta-population, EHH was measured in both directions from SNP3. In the original calculation of REHH, a particular SNP (X) was chosen so as to be 0.25 cM or 500 kb away from the core [20]. However, in the case of ST8SIA2, strong signals of selective sweep did not extend this far owing to the presence of nearby recombination hotspots. We instead defined SNP X at which the largest REHH value was observed within a region of < 500 kb and recorded the distance (*l*) from SNP3 to SNP X. To obtain the empirical distribution of REHH, SNPs of various minor allele frequencies were randomly chosen as core sites from chromosome 15 in *D*_1000_. The REHH value was calculated at a SNP site with distance *l* bp from the core site. As expected, the lower the core allele frequency, the higher the REHH value, reflecting relatively young ages of low frequency alleles and relatively high homozygosity in neighboring regions. To determine whether or not the observed REHH was an outlier, all core sites with comparable frequencies to SNP3 were selected in each meta-population (from chromosomes 3–5 and 7–22 in *D*_1000_), and ln(*REHH*) scores were examined under the assumption of their approximate normal distribution. In these REHH analyses, SNPs with minor allele frequency < 1% were discarded.

To obtain the simulated distribution of REHH under the standard neutral model with recombination, ms was performed with the command line of “./ms 1008 12000 -t 23 -r 24 18000.” For the demographic model with changing population size, fastsimcoal2 [21] was used with the specified mutation rate of μ = 1.2 × 10^−8^ per site per generation rather than the specified number of segregating sites per coalescent tree for a technical reason.

### The time duration of the operation of positive selection

Homozygote tract lengths (HTLs) [22–24] surrounding SNP3 were used to date when positive selection began to operate. As the time (*t*) back to the most recent common ancestor (TMRCA) of two homologous chromosomes increases, the HTL measured in one direction from a core site exponentially decays by recombination (with rate *r*) and mutation (with rate *μ*): The probability density *p*_1_(*x*) of *HTL* = *x* is given by

$$p_{1}(x)=2\lambda te^{-2\lambda tx}$$ (2A)

where *λ* = *r* + *μ* and the mean is given by $\bar{HTL}=1/\left(2\lambda t\right)$. On the other hand, if *HTL* is measured bidirectionally from a core site, the probability density of *HTL* = *x* becomes

$$p_{2}(x)=(2\lambda t{)}^{2}xe^{-2\lambda tx}$$ (2B)

with mean $\bar{HTL}=2/(2\lambda t)$. In this case, *t* may be estimated as $1/(\lambda \bar{HTL})$. Both *p*_1_(*x*) and *p*_2_(*x*) were used to obtain rough estimates of TMRCA. In the case where CC homozygotes at SNP3 are rare as in SAS and AMR, CC (and TT) homozygotes were generated from all pairwise comparisons of C (and T) haplotypes based on which HTLs were computed. It was assumed that *μ* = 0.5 × 10^−9^ and *r* = 1.0 × 10^−9^, both per site per year [25].

### Ancestral recombination graph in the promoter region

The 10-kb region of *D*_63_ contains 96 segregating sites and was used to demonstrate a relatively minor role of recombination in this rather restricted region. The region was also used to calibrate the TMRCA of the CGC-type and estimate the divergence times of other major haplotypes. The four-gamete test identified five haplotype blocks within the region (blocks 1–5; S3 Fig). Since blocks 1 and 2 are much larger than the remaining three, these two were used to construct gene trees by Genetree software [26] and an ancestral recombination graph by combining these trees. Block 1 (6^th^ to 41^st^) contains 27 SNPs (6^th^ to 32^nd^) in strong LD, whereas block 2 harbors the three promoter SNPs and consists of 21 SNPs (47^th^ to 67^th^). It was assumed that an effective population size (*N*_*e*_) is 10^4^ and the generation time (*g*) is 25 years.

## Results

### Geographic distribution of the CGC-type

**Fig 2:**
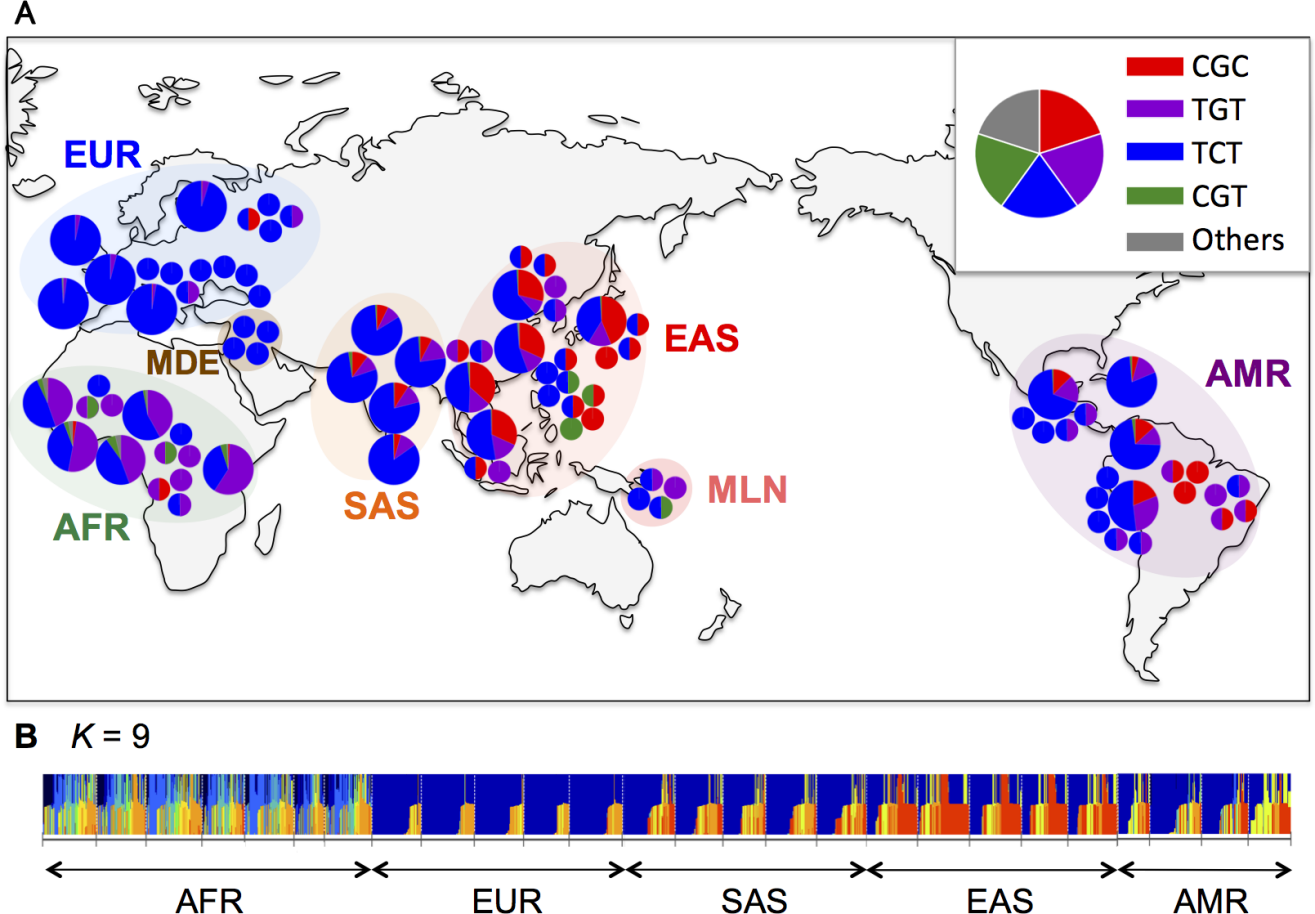
Global distribution of the *ST8SIA2* promoter types. (A) Large pie charts showing the proportion of promoter types in a population in *D*_1000_. African Caribbeans in Barbados (ACB) and Americans of African Ancestry in SW USA (ASW) are not shown because of lack of information about their homelands in Africa. Small pie charts represent individuals from 63 human samples (see S1 Table in details). (B) ADMIXTURE profile of the 18-kb region with *K* = 9 as the number of postulated ancestral populations [13] (see S4 Fig).

In the worldwide sample (*D*_1000_) taken from the present-day human population, > 99% of the *ST8SIA2* promoter is occupied by four types; TGT-, TCT-, CGT-, and CGC -types (Fig 2A; S3 Table). ADMIXTURE profiles for the 18-kb region sandwiched between the recombination hotspots show that each of the five meta-populations is quite genetically homogeneous (Fig 2B). Further increasing the number of postulated ancestral populations (*K*) does not significantly alter the profiles (see S4 Fig). The CGC-type is rare in AFR (current frequency *f* = 0.008) and almost absent in EUR (*f* = 0.002), but is relatively common in EAS (*f* = 0.35), SAS (*f* = 0.08), and AMR (*f* = 0.12) (Fig 2A). The geographic differentiation of the CGC-type moderately inflates the *F*_*ST*_ value in certain pairs of meta-populations (S4 Table), only weakly suggesting that local selection has influenced the frequency of the CGC-type. The sample size in *D*_1000_ is 1,322 for AFR, 1,006 for EUR, 1,008 for EAS, 978 for SAS, and 694 for AMR (S3 Table), indicating that the geographic heterogeneity of the CGC-type (Fig 2A) is not caused by a sampling bias. In addition, we determined all the promoter types for the 63 human samples (*D*_63_), which include many indigenous populations, to cover the local distribution of the CGC-type (Fig 2A; S1 Table). Despite the small sample size, the *D*_63_ not only confirmed the geographic heterogeneity but also revealed several additional rare variants (Fig 2A; S1 Table).

### Hitchhiking of neighboring SNPs by the increase of the CGC-type frequency

**Fig 3:**
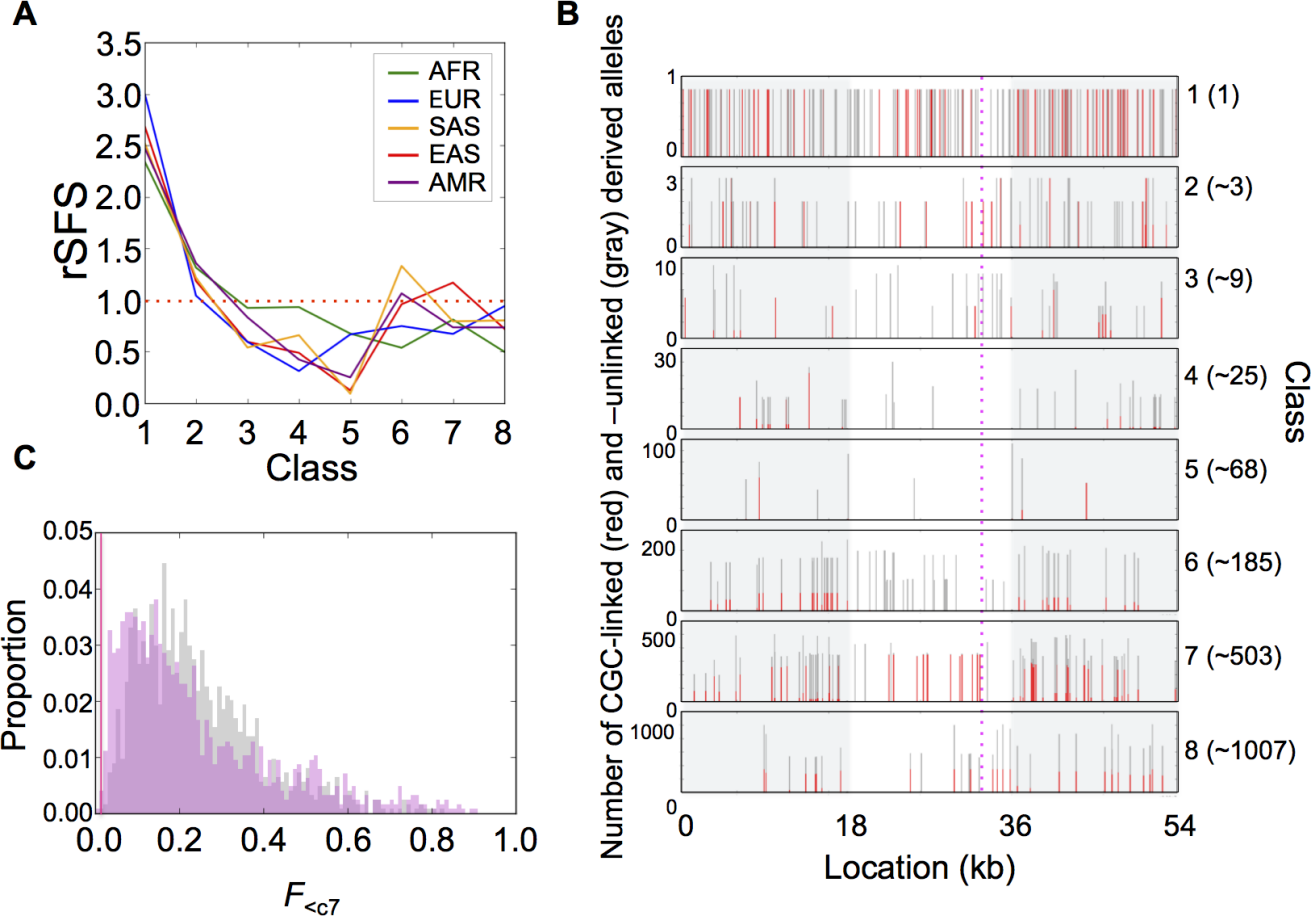
Site frequency spectrum and barcode representation. (A) Relative site frequency spectrum (rSFS) that is defined as the ratio of observed-to-expected proportion of SFS in the 54-kb region. The rSFS is calculated from *D*_1000_ and the expectation under the standard neutral model of constant size. (B) Barcode representation of SNPs in the 54-kb region of EAS. The red dotted line shows the location of the three promoter SNPs. A red bar and a gray bar at an individual SNP site indicate the number of derived alleles that are linked to the CGC-type and the nonCGC-type, respectively. The unshaded area corresponds to the 18-kb core LD region. (C) Simulated distributions of *F*_<c7_ under the standard model of constant size (1,237 replications in gray) and the demographic model of changing population size (1,259 replications in magenta; [17]). The red vertical line represents the observed *F*_<c7_ in EAS.

We first examined the 54-kb region for the SFS (Fig 1). As expected, all the meta-populations exhibit substantial excess of rare alleles in *D*_1000_ (Fig 3A). The excess is consistent with a recent population expansion and may well be explained by human demographic models that incorporate such an effect (e.g., [17]). In EAS, SAS, and AMR, Tajima’s D (*P* = 0.13−0.40; [27]) and Fay and Wu’s H (*P* = 0.16−0.29; [28]) are only moderately negative (S5A Table). This remains unchanged even if the more tightly linked 18-kb core region is examined (S5A Table). Interestingly, however, the G-test by comparing observed and expected/simulated SFSs showed significantly high levels of observed intermediate allele frequencies (*P* < 0.01; Fig 3A; S5 Fig).

To test the possibility that natural selection has indeed favored the CGC-type, we analyzed relative SFS (rSFS) more carefully and found increased allele frequencies (rSFS > 1) in class 7 of EAS and in class 6 of SAS or AMR (Fig 3A and S5 Fig). We confirmed that these increases result from increased numbers of derived alleles preferentially linked to the CGC-type. For instance, in the 54-kb region of EAS, 42% (12,312 out of 29,184) of derived alleles in class 7 are linked to the CGC-type, which is 1.2-times higher than expected ($\chi_{d.f.=1}^{2}$ = 430.7). The same preferential association occurs in class 6 of SAS (14% with $\chi_{d.f.=1}^{2}$ = 475.7; 1,459 out of 10,381) and AMR (17% with $\chi_{d.f.=1}^{2}$ = 234.9; 1,744 out of 10,089).

### Detecting positive selection acting on the CGC-type by a new method

We applied a newly developed statistical method that visualized the pattern and level of variability in a core region by the barcode representation, and quantified the IAV within an allele group. The barcode representation in EAS shows 12 prominent red bars in class 7 (corresponding to 12 SNP sites with derived alleles that are linked to the CGC-type), and conversely shows marked deficiency in classes 4–6 (Fig 3B). As the copy number of the CGC-type is 349 in EAS, the core frequency belongs to class 7. It turns out that the estimated value of *F*_<c7_ is only 1.5%, indicating that among all derived alleles in classes 1–6, only a small number of derived alleles have accumulated within the CGC group. All simulations for neutral mutations failed to explain this low level of *F*_<c7_ (*P* = 0.0019 under the standard model and *P* = 0.0025 under the demographic model; Fig 3C). Simulation thus demonstrated a low false positive rate or a low type I error of the *F*_*c*_ statistic. It is also worthy to note that nonCGC-types did not show any significantly low level of *F*_c_ (*F*_<c8_ = 0.39, *f*_*r*_ = 0.65, *P* > 0.40).

### Detecting positive selection acting on the CGC-type by REHH

**Fig 4:**
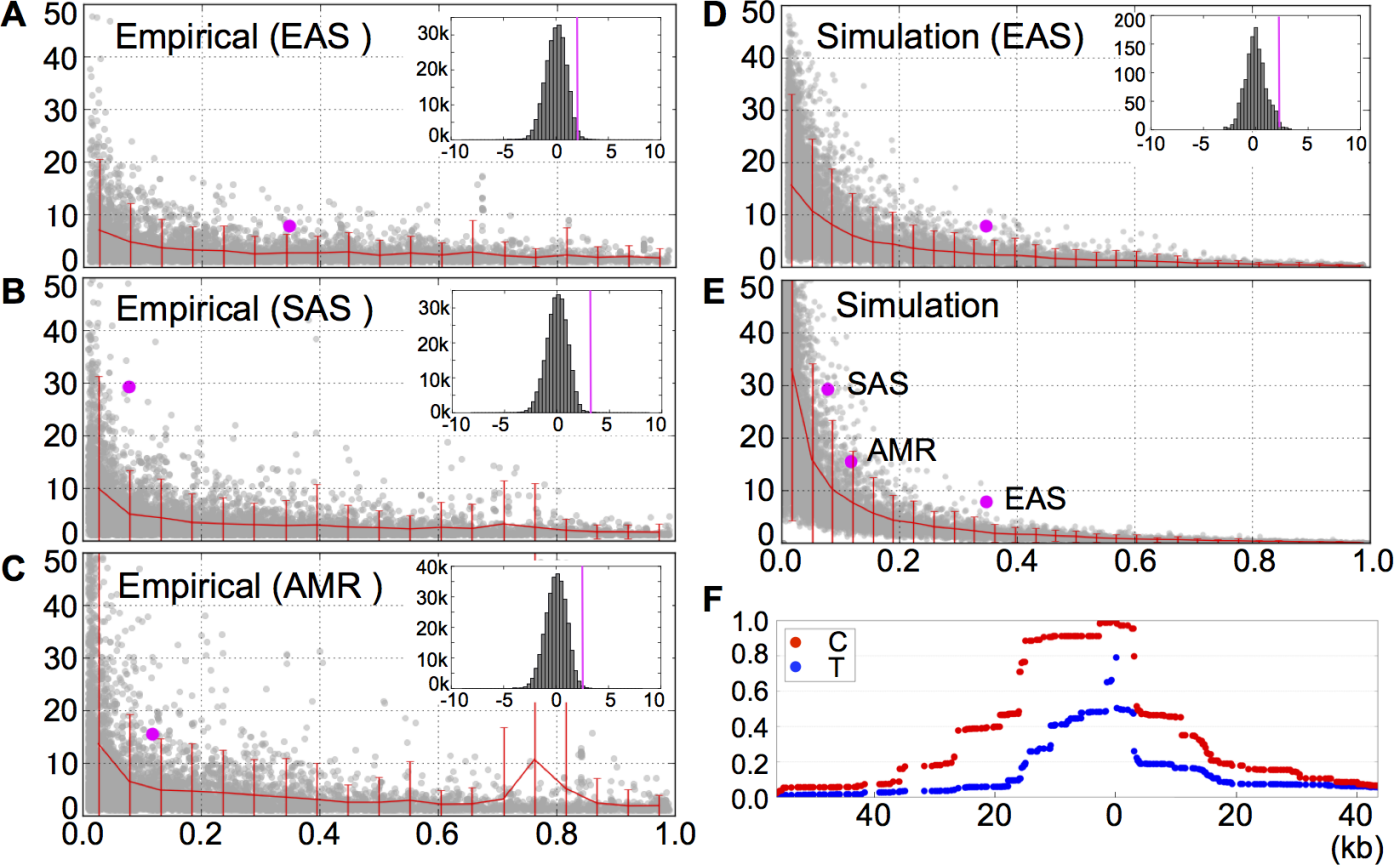
Relative extended haplotype homozygosity (REHH) of the CGC-type in EAS, SAS and AMR. REHH values are plotted against core allele frequencies. (A**–**C) Observed REHH values (magenta dots) and empirical distributions in chromosome 15 for EAS, SAS, and AMR. For a given frequency at a core SNP, red lines indicate the 95^th^ percentile. Each inset depicts the empirical distribution of standardized ln(*REHH*) for genome-wide SNPs with allele frequencies comparable to the CGC-type (chromosomes 3–5 and 7–22). The magenta lines indicate the observed values. (D) The observed REHH value for EAS (magenta dot) together with the simulated distribution under the demographic model of changing population size [17] (10,000 replications). The inset shows the standardized ln(*REHH*) distribution for SNPs with derived allele frequencies comparable to the CGC-type. Simulation is based on 1,200 replications (the observation is indicated by the magenta line). (E) Observed REHH values for EAS, SAS, and AMR (magenta dots) together with the distributions simulated by ms (under the standard neutral model with 10,000 replications). (F) Decay of EHH from SNP3 (at 0 location) in EAS.

Like other LRH (Long Range Haplotypes) and our *F*_*c*_ statistic, REHH is sensitive to the current frequency *f* of a focal allele [29, 30]. The observed largest REHH value near the core site is 8 in EAS, 29 in SAS, and 16 in AMR, indicating less breakdown of homozygosity in the CGC-type than the nonCGC-type. Despite the obvious *f*-dependence, these upward deviations of all the three observed REHH values are statistically significant in comparison with the empirical distribution (Fig 4A–4C) and the simulated distribution (Fig 4D and 4E). The mean and standard deviation for empirical ln(*REHH*) scores are 0.286 and 0.859 for EAS, 0.358 and 0.861 for SAS, and 0.340 and 0.920 for AMR. The standardized empirical score is 2.07 for EAS (*P* < 0.02), 3.51 for SAS (*P* < 0.0003), and 2.61 for AMR (*P* < 0.005). Although our way of detecting positive selection through REHH is based on the largest value in a specified genomic region and thus different from the original method [20], both simulation and empirical distributions of REHH supports that the CGC-type inscribes a significant signature of selective sweep. This is consistent with the high EHH of the CGC-type even across recombination hotspots (Fig 4F).

### Dating the action of positive selection on the CGC-type

We used homozygosity tract lengths (HTLs) [22–24] to estimate the time (*t*) elapsed since positive selection began to operate on the CGC-type. In the pairwise comparison of all CGC haplotype sequences, the mean of bidirectional HTLs is 28 kb for EAS, 27 kb for SAS, and 35 kb for AMR (S6 Table). Substituting these mean values for formula (2b), we obtained *t* as 24, 25, and 19 thousand years (ky) for EAS, SAS, and AMR, respectively. However, recombination hotspots are located near SNP3 (Fig 1) with the right hotspot being much closer to the core site than the left. For this reason, the above *t* values may be overestimates (Fig 1; S6 Table). If we instead use only the left HTL of SNP3, formula (2a) yields the time as 19 ky for EAS, 20 ky for SAS, and 18 ky for AMR.

### Evolutionary history of promoter types and ongoing selective sweep by the CGC-type

**Fig 5:**
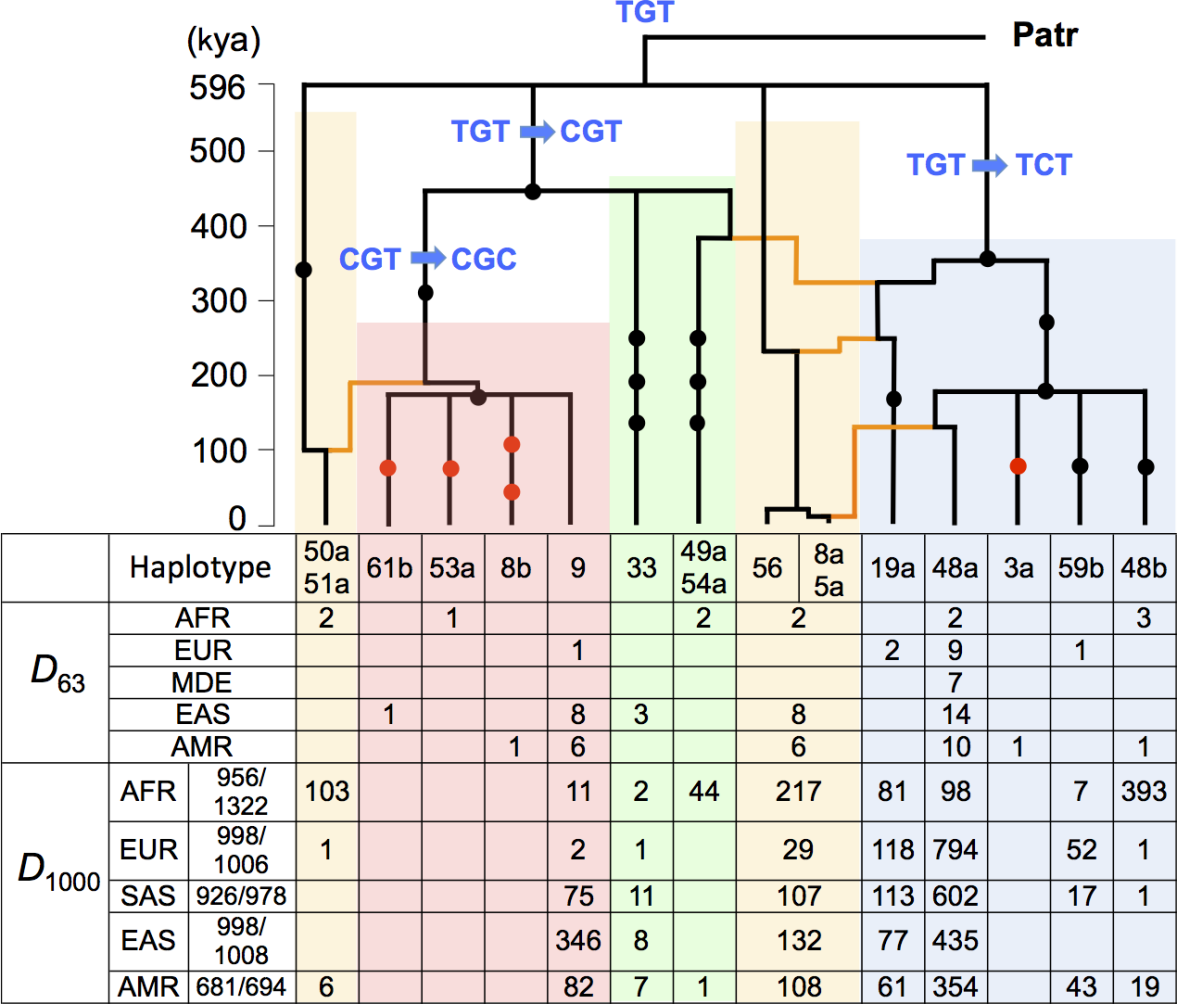
Ancestral recombination graph in the human *ST8SIA2* promoter region. Ancestral recombination events in the 10-kb core LD region are inferred by comparing tree topologies between two neighboring blocks 1 and 2 (S6 Fig and S7 Table). The gene trees for blocks 1 and 2 are drawn simultaneously with required recombination events (orange lines). Dots on branches represent SNPs in block 2, of which five SNPs shown by red dots are present in *D*_63_ but absent in *D*_1000_. The number of each haplotype of block 2 is summarized with their geographic distribution in *D*_63_ and *D*_1000_.

We determined the 10-kb haplotype sequences in 63 human samples coming from many indigenous populations (Fig 1 and S7 Table). As aforementioned, this sequence dataset (*D*_63_) contains additional information on the geographic differentiation of the CGC lineage (Fig 2A and S8 Table) as well as rare CGC haplotype sequences that are not found in *D*_1000_ (Fig 5). Here we used *D*_63_ to estimate the divergence times of the CGC lineage and other lineages. First, using the four-gamete test, we divided the 10-kb region into five haplotype blocks (S3 Fig). Genetree analysis of 91 haplotype sequences in Fig 5 suggests that the CGC lineage diverged from the CGT lineage and began to further diversify 180 kya. Furthermore, the time back to the most recent common ancestor (TMRCA) of all distinct lineages is estimated as 596 kya, whereas the divergence time of the TCT and CGT lineages is estimated as 359 kya and 455 kya, respectively. All nonhuman primates examined thus far (six chimpanzees, 14 gorillas, and other nonhuman primates) have only TGT haplotype sequences (Fig 5). In the ancestral recombination graph, only the CGC haplotype sequences are tightly clustered together even in different LD blocks 1 and 2 (Fig 5 and S6 Fig). This observation further supports the view that the CGC lineage has expanded so rapidly that recombination did not have enough time to shuffle the cluster genealogically (see below).

Based on the number of accumulated mutations within the CGC cluster in blocks 1 and 2, the maximum likelihood estimation [31] shows that the CGC lineage began to diversify into sub-lineages between 100–400 kya. Hence, the mutational diversification of the CGC lineage is much older than the HTL-based age estimate (20–30 kya), the latter being regarded as the time when the selective sweep began to take place by a small number of founding CGC lineages. Moreover, as the CGC lineage is maintained in the Mbuti Pygmy population, a basal group [32] of the phylogeny of anatomically modern humans (AMHs) (haplotype 53a; Fig 5; S1 and S8 Tables), the CGC lineage had likely been maintained in AFR for a long time as a standing variation before the action of positive selection. Nonetheless, the CGC lineage is still confined in some geographic regions and segregating in intermediate frequencies, which also supports the short history of selection on this lineage. Thus, positive selection on the CGC-type is still ongoing and has conferred soft selective sweep of linked neutral polymorphisms.

### Promoter activity of *ST8SIA2* gene in humans and great apes

**Fig 6:**
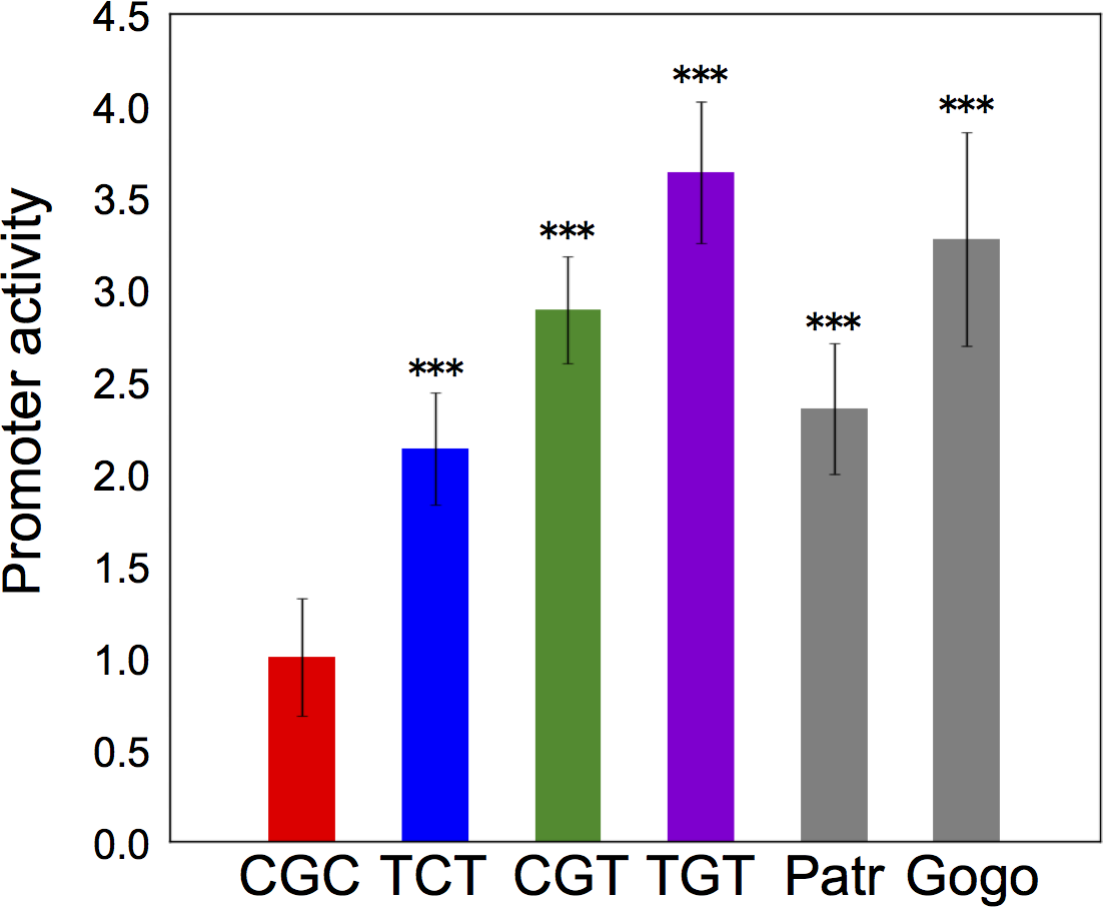
Promoter activities of the human, chimpanzee and gorilla *ST8SIA2* promoter types measured by luciferase expression. Each value represents mean ± standard error of the mean over six independent transfection experiments. Data are represented as relative fold-increase compared with the human CGC-type. Chimpanzee (Patr) and gorilla (Gogo) possess the TGT-type promoter.

The promoter activity of the CGC-type was previously shown to be lower than that of the TGT-type [7]. However, the TCT-type is most prevalent in non-African populations (Fig 2). In addition, the CGC-type may have competed and gained selective advantage against the TCT-type, particularly in EAS. It is therefore essential to further investigate the promoter activities of all four types (i.e., TGT-, TCT-, CGT-, and CGC-types). The promoter activity is significantly lower only in the CGC-type (*P* < 0.005, Fig 6). It is also noteworthy that the promoter activities of the TCT-, CGT-, and human TGT-types are comparable to the African ape TGT-type. Furthermore, unlike the CGT-type that is shared with Neanderthals (data not shown), the CGC-type is of relatively recent origin and most likely unique to AMH. These indicate that the low expression level of *ST8SIA2* has been favored by natural selection under certain genetic and environmental circumstances during the range-expansion of AMH in the Upper Paleolithic and Neolithic era.

## Discussion

By definition, ongoing positive selection does not necessarily accomplish dramatic elevation of allele frequencies at its target site as well as linked neutral sites. This moderates genetic differentiation among populations and thereby markedly reduces the statistical power of any method that relies on such differences. In this study, we could not detect any significant signals of positive selection on the CGC-type using *F*_*ST*_ and similar statistics. Likewise, other methods based on SFS (or its summaries) and LRH (such as iHS [33]) have also failed to detect convincing signals of positive selection on the CGC-type of the *ST8SIA2* gene. It is certain that the main reason for the weak power of SFS-based methods resides in the fact that the population frequency of the CGC-type is at most 35% in EAS and even lower in SAS and AMR. By contrast, it is likely that the weak power of LRH-based methods is attributed to the close proximity of the three promoter SNPs to the recombination hotspots: these methods are sensitive to recombination rates and may be easily obscured by their enhancement. Nonetheless, our approach which modified the REHH has identified a signal of positive selection.

Under these circumstances, development of a new method is still preferable for detecting ongoing selective sweep bounded by nearby recombination hotspots. Our new method is based on the barcode representation of both SFS and LD information as well as on quantification of IAV. The barcode representation facilitates in identifying a core region that includes a putative target site (a core site) of positive selection and is in strong LD with the core site. To measure the IAV within an allele group of interest, we have defined in formula (1) the *F*_*c*_ statistic as the proportion of derived alleles that occur in the derived allele group specified at the core site. In computing the statistic, it is essential to exclude certain frequency classes of derived alleles that have accumulated in basal branches in a genealogical tree. The *F*_*c*_ statistic restricts its application to such regions that do not contain recombination hotspots, yet have a sufficiently large number of segregating sites. In the case of *ST8SIA2* in EAS, there are 160 SNPs in the 18 kb LD region. Simulation studies with and without positive selection have shown that these numbers are sufficiently large for the *F*_*c*_ statistic to capture ongoing sweep signals.

*ST8SIA2* is of vertebrate origin and as a gene involved in brain function, homologs can be found in all vertebrate genomes thus far studied. At the protein sequence level (375 amino acids), the gene is evolutionarily conserved and we calculated the ratio of nonsynonymous to synonymous substitutions per site between humans and mice to be only 0.033, much lower than 0.3 of ß-hemoglobin. In the comparison of closely related species such as humans and chimpanzees, we found only two nonsynonymous substitutions and within humans, no such substitutions are segregating. These general features are consistent with the functional importance of the gene and may well be accounted for by negative selection against qualitative changes of the gene.

In addition to the three promoter SNPs, four SNPs are found in the sequences used for the promoter assay (see S7 Table). Thus, the detected difference in promoter activities (Fig 6) might be interpreted by the combination of these seven SNPs. However, since the major sequence of each promoter type was used for the promoter assay (see S7 Table), the result in Fig 6 reflects the reality of promoter activity polymorphism in the human populations. Our results of *ST8SIA2* promoter activity have indicated that quantitative changes of the gene product are a target of positive selection. Although the complete lack of the gene product is presumably deleterious in mice [5, 6] due to its functional importance, it is still conceivable that CGC homozygotes with lowered levels of the gene product are more fit and advantageous than nonCGC homozygotes with heterozygotes being intermediate. In this case, positive selection operates in a form of directional selection. An alternative possibility may lie in genotype-environment interactions. Since it is most likely that mental activity is a quantitative trait that is controlled by an orchestration of many genes [34–36], variation at individual genes involved may be maintained by genotype-environment interactions through a concave fitness function of a causal variable *x* (e.g., enzyme activity) [15, 37, 38]. If the CGC-type and nonCGC-type both are expressed in a manner of semidominance, *x* or the amount of ST8SIA2 produced by heterozygotes would have intermediate *x* values between that of CGC and nonCGC homozygotes. The concave relationship between fitness and *x* as well as assumed heterogeneous environments then can lead to higher mean fitness of heterozygotes relative to homozygotes [37]. Clearly, such a relationship results in a form of balancing selection, but it resembles directional selection can act when the frequency of the CGC-type is low.

The contribution of each gene to a quantitative trait should be variable, depending on its role in an orchestration. Operation of positive selection on the CGC-type raises the possibility that *ST8SIA2* is a promising target in understanding schizophrenia development. A potential molecular mechanism for the risk avoidance may be functionally associated with appropriate PSA expression. The positive selection on the CGC-type suggests that low amounts of ST8SIA2 protein are advantageous under some circumstances. This may imply that low amounts of PSA are a target of the positive selection acting on the CGC-type (but see [39] for PSA reduction in patients). Interestingly, the *ST8SIA2* gene is involved in not only schizophrenia but also bipolar disorder and autism [2]. Two intronic SNPs that are associated with bipolar disorder and autism, respectively, affect the expression of pre-mRNA and mRNA, and alter the cellular levels of ST8SIA2 and PSA [40]. Taken together with the finding that the promoter activity of *ST8SIA2* is involved in schizophrenia (Fig 6), it appears that transcriptional change of *ST8SIA2* has an impact on mental activities.

The ancestral promoter type of the *ST8SIA2* gene in the human lineage was originally TGT, as found in non-human primates, from which the TCT- and CGT-types near-simultaneously descended about 600 kya (Fig 5). This estimated emergence time of the CGT-type is consistent with the finding that Neanderthals and Denisovans possessed the CGT-type, provided that these archaic humans diverged from the ancestral lineage of AMHs 550–765 kya (S7 Fig) [41]. The AMH-specific CGC lineage originated about 400 kya and further diversified into sub-lineages within African populations, although their frequencies have remained low till the present day. It is not known when and in which route(s) the CGC lineage migrated out of Africa and spread in Eurasia. The present-day distribution of the CGC lineage in Eurasia provides a contrasting pattern between the almost complete absence in the West and the moderate commonness in the East. It appears that none of the three or more European genetic components [42, 43] brought the CGC lineage into Europe. If this conclusion is also applied to the first AMH that lived in Europe, it becomes likely that ancestral Eastern Eurasians around 36–45 kya did not harbor the CGC lineage either. The Simons Genome Diversity Project database supports this conjecture by exhibiting localization of the CGC lineage (data not shown).

We have shown that positive selection began to act on the CGC promoter type in Asia since around the Last Glacial Maximum (LGM; 19–26.5 kya; [44]). The LGM is delineated as a critical phase in biological and cultural evolution of Upper Paleolithic AMH populations, and had a profound impact on the human lifestyle and behavior [45]. Since the LGM, AMHs have improved their skills and technologies to survive against various environmental challenges, finally reaching the agricultural revolution in the Neolithic stage. Several mass admixture events occurred in Asia during the range-expansion of AMHs across the Eurasian continent (S8 Fig). One occurred in South Asia and Southeast Asia at least 25 kya, between people already settled by the earlier migration into the southern part of Eurasia (earlier south migrants) and those who migrated much later (later south migrants) [46]. Another admixture event occurred in the eastern part of Eurasia after the LGM, which was between the later south migrants and those who reached into North Asia via Central Asia by 27 kya [47]. Therefore, in Asia, the prehistoric progress occurred since around the LGM with drastic changes in social environments such as intragroup organization and intergroup interaction. Present-day populations showing a selective sweep by the CGC-type were established as a consequence of these migrations and admixtures (S8 Fig). Psychosocial stress is a major environmental risk factor involved in the onset of schizophrenia [48–50], and may have arisen from tension during adaptation to changing social environments in Asia. Although other environmental risk factors such as winter birth were well-known, we may therefore regard psychosocial stress as a crucial selective pressure on the CGC-type and presume that the CGC-type can confer tolerance under changing social environments. Positive selection on the CGC-type thus raises a possibility that mental adaptation or adaptation in mental activities has occurred since the LGM. If the migrants had different culture with each other, positive acceptance and learning of different culture might induce improvement of survival skills by the admixture. Psychosocial stress arising from tension during adaptation to an alien culture, namely acculturative stress, is suggested to be an important environmental risk factor in development of the mental disease [51]. The CGC-type might confer acceptance to cultural differences by tolerance for acculturative stress, and contribute to enable people to be open-minded to changing their own culture by learning from a different one. This might be an important function in mental adaptation to changing social environments since the LGM.

The later south migrants seem to have contributed to the genetic diversities of present-day East Asian populations more than the earlier south migrants [46]. Since the basal sequences (HG00419.1 and HG03809.1) in the CGC haplotype tree are found exclusively in EAS and SAS (S9 Fig and S9 Table), the CGC-type might have been brought into these areas by the later south migrants. In addition, Druze samples from the Middle East (MDE; Fig 2A) also have no CGC-type (S1 and S8 Tables), suggesting that geographic heterogeneity in CGC-type frequency emerged after the eastward migration from West Eurasia. These are consistent with our dating of the CGC-type mediated selective sweep.

In conclusion, we found that a non-risk type of schizophrenia development, namely the CGC-type, has been selected mainly in Asia since the LGM, and its promoter activity is significantly lower than those from risk types. These suggest that quantitative changes of ST8SIA2 protein are a selective target under changing social environments in post-glacial Asia. Schizophrenia becomes clinically evident by environmental risk factors such as psychosocial stress. The positive selection on the CGC-type in EAS, SAS, and AMR suggests that environmental risk factor (selective pressure) prevailed since the LGM and has caused schizophrenia by affecting mental activities during the Upper Paleolithic and Neolithic era. Based on this, we proposed a possible scenario, mental adaptation to changing social environments, to explain the evolutionary background of the positive selection on the CGC-type. This might also indicate the recent origin of schizophrenia, a possible evolutionary time-frame to explain why highly heritable schizophrenia is so prevalent in present-day human populations. Many genes are associated with schizophrenia [34–36]. We could not obtain direct evidence for positive selection of the CGC-type in EUR and AFR, but our findings raise the possibility that positive selection at other schizophrenia-related loci may be detected in EUR and AFR as well. From this, understanding of the evolutionary basis of schizophrenia prevalence would be then deepened, shedding more light on the significance of mental adaptation in the evolution of AMHs.

## Supporting information

S1 Fig Estimated mean recombination rates in CHB, JPT, CEU, and YRI populations. Recombination rates were calculated by the LDhat 2.2 program using Han Chinese in Beijing, China (CHB), Japanese in Tokyo, Japan (JPT), Utah Residents (CEPH) with Northern and Western European Ancestry (CEU), and Yoruba in Ibadan, Nigeria (YRI) populations of *D*_1000_.
(PDF)

S2 Fig Genealogy revealed by barcode representation in the 18-kb region. Mutations belonging to each class are assigned to each colored branch.
(PDF)

S3 Fig Matrix of four gamete test. Among the 96 SNPs in *D*_63_, we placed SNP sites that are shared by more than two *D*_63_ haplotypes and are not compatible to all sites. The resulting 49 SNP sites were used for the four-gamete test. The pair of sites under a linkage break is represented by an asterisk. Five haplotype blocks were identified. The positions of the three promoter SNPs are highlighted by yellow.
(PDF)

S4 Fig ADMIXTURE analysis. (A) ADMIXTURE pattern is not changed by increasing number of postulated ancestral populations (*K*). (B) ADMIXTURE pattern (*K* = 9) sorted by promoter types shows that the CGC type is homogeneous. (C) Cross validation error does not change with *K* ≥ 9.
(PDF)

S5 Fig Relative site frequency spectrum (rSFS) in the 54-kb region. rSFS was defined as the ratio of observed-to-simulated proportions of SFS under the demographic model [17] using *μ* = 1.2 × 10^−8^ per site per generation.
(PDF)

S6 Fig Neighbor-joining trees for the five haplotype blocks in *D*_63_. Neighbor-joining trees were constructed using the five haplotype blocks detected by the four-gamete test in *D*_63_. The sequences of each promoter type are highlighted by colors [TGT-type (purple), TCT-type (blue), CGT-type (green), and CGC -type (red)].
(PDF)

S7 Fig Promoter type of archaic humans. The emergence time of the CGC-type lineage is estimated to 455 thousand years ago (kya) (Fig 5), which is much later than the time of the population split of archaic humans from anatomically modern humans (AMHs) (550–765 kya; [41]). Moreover, the CGC-type has not been identified in archaic human genomes (data not shown). This indicates that the CGC-type emerged uniquely in AMHs. Recently, it has been reported that adaptive haplotypes were introduced from archaic humans to AMHs by introgression [24, 52]. However, the selective sweep by the CGC-type does not show this. Promoter type identified from a single individual known as Denisovan, an archaic human who lived in Asia, is classified as a member of the CGT-type. In addition, two Neanderthal individuals (Vindija and Altai) are homozygous for the CGT-type (data not shown), which implies that the frequency of the CGT-type in archaic humans might be considerably higher than in AMHs (1.5% in *D*_1000_).
(PDF)

S8 Fig Demographic events involved in the selective sweep by the CGC type. In the out-of-Africa migration, anatomically modern humans (AMHs) migrated into the Eurasian continent by three major dispersals [42, 43]. The north dispersal reached into North Asia via Central Asia by 27 thousand years ago (kya) [47], while the other two other dispersals (earlier and later south dispersals) occurred at different times in the same route passing through South Asia into Southeast Asia. (A) In the earlier south dispersal, people finally migrated into the Australian continent from Southeast Asia at least 50 kya or possibly 65 kya [53]. (B) In the later south dispersal, people finally migrated into East Asia from Southeast Asia at least 25 kya [46]. During this later south dispersal, massive admixture occurred between the people already settled in South Asia and Southeast Asia by the earlier south dispersal (earlier south migrants) and those migrated by the later south dispersal (later south migrants) [46]. The people that migrated into North Asia by the north dispersal (north migrants) and later south migrants had been unable to migrate further because of cold environments that appeared during the Last Glacial Maximum (LGM). (C) After the LGM, they started moving extensively northward and southward in the eastern part of Eurasia [54–60]. These migrations caused frequent close encounters between later south migrants and north migrants, and resulted in massive admixture in the eastern part of Eurasia, as shown by the unique genetic structure (i.e., dual genetic structure) of East Asian populations from mitochondrial genome and Y chromosome analyses [54, 61]. (D) The people who underwent admixture in the eastern part of Eurasia, simultaneously migrated into the American continent with the appearance of the Bering land bridge (Beringia) around 15 kya. This is suggested by the finding that Native Americans have mixed origins resulting from admixture between people related to East Asians and Western Eurasians [62]. Thus, present-day populations showing the selective sweep by the CGC-type (i.e., EAS, SAS, and AMR) were established in massive admixtures that occurred after the LGM.
(PDF)

S9 Fig A neighbor-joining tree for all the CGC haplotype sequences. A neighbor-joining tree was constructed using the SNP data of *D*_63_ and *D*_1000_ with MEGA7 software [63]. The overlapped part (9 kb) between the 10-kb and the 18-kb regions was used (Fig 1; S7 and S8 Tables). A TGT haplotype (11-a) was used as an outgroup. Total 47 segregating sites were involved. The percentage of replicate trees in which associated taxa clustered together in the bootstrap test (1,000 replicates) is shown next to the branches. The tree is drawn to scale, with branch lengths in the same units as evolutionary distances used to infer the phylogenetic tree. Evolutionary distances were calculated using the number of differences method, and are in units of number of base differences per sequence.
(PDF)

S1 Table Promoter types for the 63 human samples. All 63 samples are listed with IDs, and the meta- and sub-populations to which they belong, repository numbers at Coriell Cell Repositories, and genotypes at the three promoter SNPs.
(PDF)

S2 Table Primer sequences. The sequences of primers used in this study are listed.
(PDF)

S3 Table Promoter types frequencies in the *D*_1000_ sequence dataset. (PDF)

S4 Table *F*_ST_ values for SNPs in the *ST8SIA2* promoter region. *F*_ST_ values were calculated for SNPs in the 54-kb region between meta-populations. Mean (mean *F*_ST_), standard deviation (Std of *F*_ST_), and maximum values (maximum) of *F*_ST_ are shown with the number of segregating sites (*S*) and 90^th^ and 95^th^ percentiles of *F*_ST_. *F*_ST_ values for the three promoter SNPs (SNP1–3) were also calculated. *F*_ST_ values highlighted in salmon pink are greater than the 95^th^ percentile, while those in orange are greater than the 90^th^ percentile.
(PDF)

S5 TableGenetic variability in the *ST8SIA2* locus.(A) Genetic variability in three regions in *D*_1000_. The 54-kb region was separated into regions A, B and C of each 18 kb length. Region B corresponds to the 18-kb region (Fig 1) that spans the three SNPs and sandwiched between weak recombination hotspots. (B) Genetic variability in *D*_63_. Note: ^a^The expected haplotype (allele) number in a sample of *n* chromosomes with estimated *θ*_*w*_ and *θ* values under the assumption of no recombination within each region [64]. In an equilibrium population of effective size *N*_*e*_, both *θ*_*w*_ and *θ* reflect scaled-per-site neutral mutation rate (4*N*_*e*_*μ*) estimated from the number of segregating sites (*S*_*n*_) or nucleotide diversity (*π*). ^b^Tajima’s D and ^c^Fay and Wu’s H were calculated using Dnasp. Statistical significance was also assessed based on 1,000 simulations using Dnasp under free recombination (for *D*_1000_) or mean recombination rate in this region (for *D*_63_) calculated by LDhat (*ρ* = 4*N*_*e*_*r* = 13.2, where *N*_*e*_ represents effective population size and *r* recombination rate per gene). Tests marked with asterisks were significant (*P* < 0.05). ^d^Squared correlation coefficient is approximated as *r*^2^ = *D*^2^/(*p*^*A*^*q*^*A*^*p*^*B*^*q*^*B*^), where *p*^*A*^ and *p*^*B*^ are allele frequencies at sites A and B, respectively, and *p*^*A*^ + *q*^*A*^ = *p*^*B*^ + *q*^*B*^ = 1. The average squared correlation coefficient was calculated from all pairs of polymorphic sites within a region in each population. The scaled recombination rate, *ρ* [65, 66], was calculated from *r*^2^ using $E(r^{2})\approx \sigma_{d}^{2}\approx \frac{10+\rho }{22+13\rho +\rho^{2}}\approx \frac{10+\rho }{\left(11+\rho )(2+\rho \right)}\approx \frac{1}{2+\rho }$. Values of *χ*^2^ = *kr*^2^ were no smaller than 10 [with a minimum value of 13.2 in Region C of Africa (AFR)], implying significant linkage disequilibrium (*P* < 0.0003) in each population despite *H*_*n*_ >> *E*(*H*_*n*_). *P < 0.05.(PDF)

S6 Table Homozygosity tract length (HTL) in meta-populations. HTLs for CC and TT homozygotes are shown together with the number of tracts (number of homozygous individuals). Total HTL and HTL in the left-side and right-side of cores were measured separately. HTL was also calculated by pairwise comparison using all chromosomes within a meta-population.
(PDF)

S7 Table Alignment of 96 segregating sites in 91 haplotype sequences of *D*_63_. Segregating sites were compared with nucleotides at corresponding sites of primates [Chimpanzee (Patr) and Gorilla (Gogo)]. Dots refer to nucleotides that are identical with the chimpanzee sequence. The three promoter SNPs (SNP1–3) are highlighted in yellow. The 21 SNPs not found in *D*_1000_ (newly discovered SNPs) are highlighted in pink. The haplotypes used for the promoter assay are marked with blue. The locations of the overlapped region with the 18-kb region, Block 1–5, and the region used for the promoter assay are shown on top of the table.
(PDF)

S8 Table Distributions of the CGC-type in *D*_63_. Distribution of the 6 CGC haplotypes in *D*_63_ are shown. The haplotypes are defined using the overlapped part (9 kb) between the 10-kb and the 18-kb regions.
(PDF)

S9 Table Distributions of the CGC type in *D*_1000_. Distribution of the 31 CGC haplotypes in *D*_1000_ are shown. The haplotypes are defined using the overlapped part (9 kb) between the 10-kb and the 18-kb regions.
(PDF)
