# Supplementary figures and images for: Positive selection on schizophrenia-associated ST8SIA2 gene in post-glacial Asia
Source: PLoS One. 2018 Jul 25;13(7):e0200278. doi: 10.1371/journal.pone.0200278 (PMC6059407; doi:10.1371/journal.pone.0200278)

S1 Fig. Estimated mean recombination rates in CHB, JPT, CEU, and YRI populations.

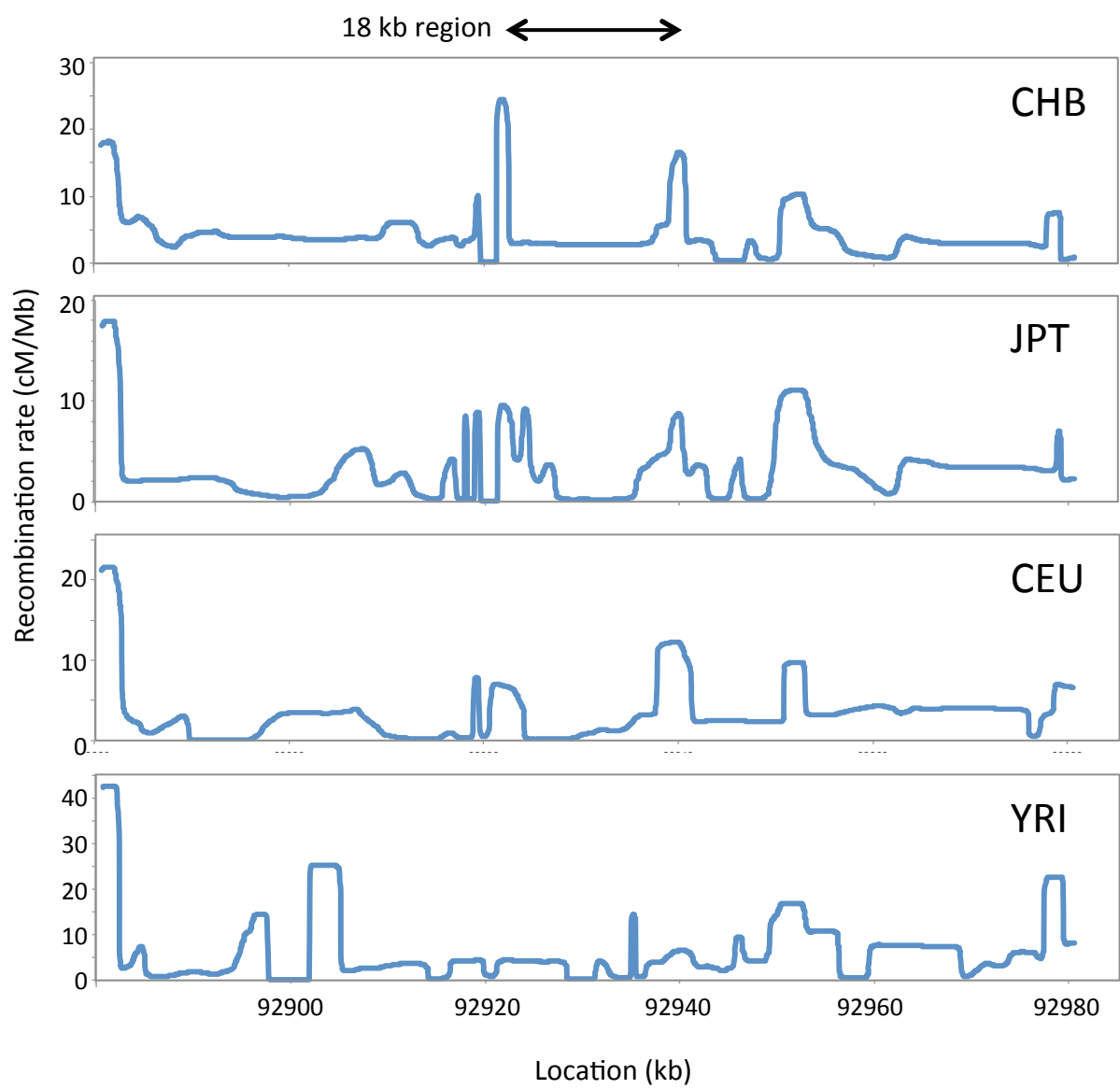

Supplement: S1 Fig — Recombination rates were calculated by the LDhat 2.2 program using Han Chinese in Beijing, China (CHB), Japanese in Tokyo, Japan (JPT), Utah Residents (CEPH) with Northern and Western European Ancestry (CEU), and Yoruba in Ibadan, Nigeria (YRI) populations of D1000. (PDF) [file pone.0200278.s001.pdf]

S2 Fig. Genealogy revealed by barcode representation in the 18-kb region.

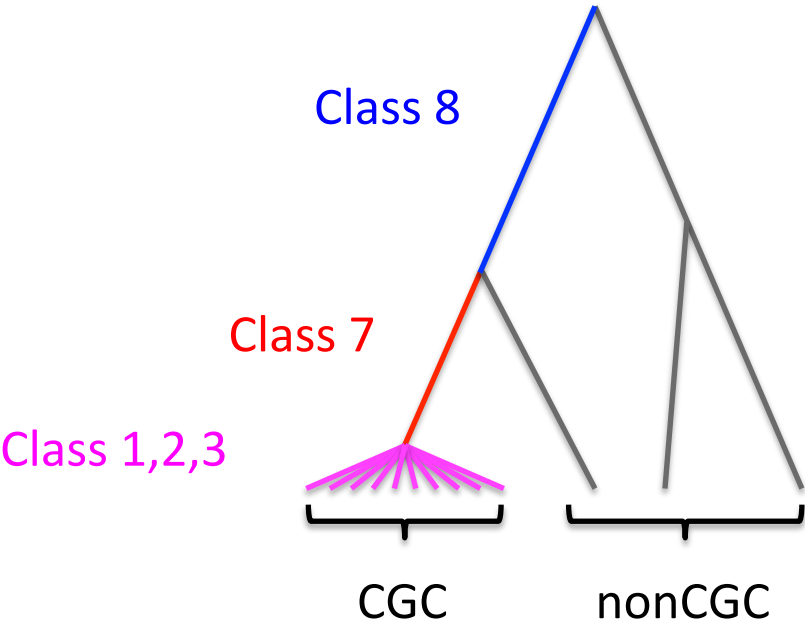

Supplement: S2 Fig — Mutations belonging to each class are assigned to each colored branch. (PDF) [file pone.0200278.s002.pdf]

S3 Fig. Matrix of four gamete test.

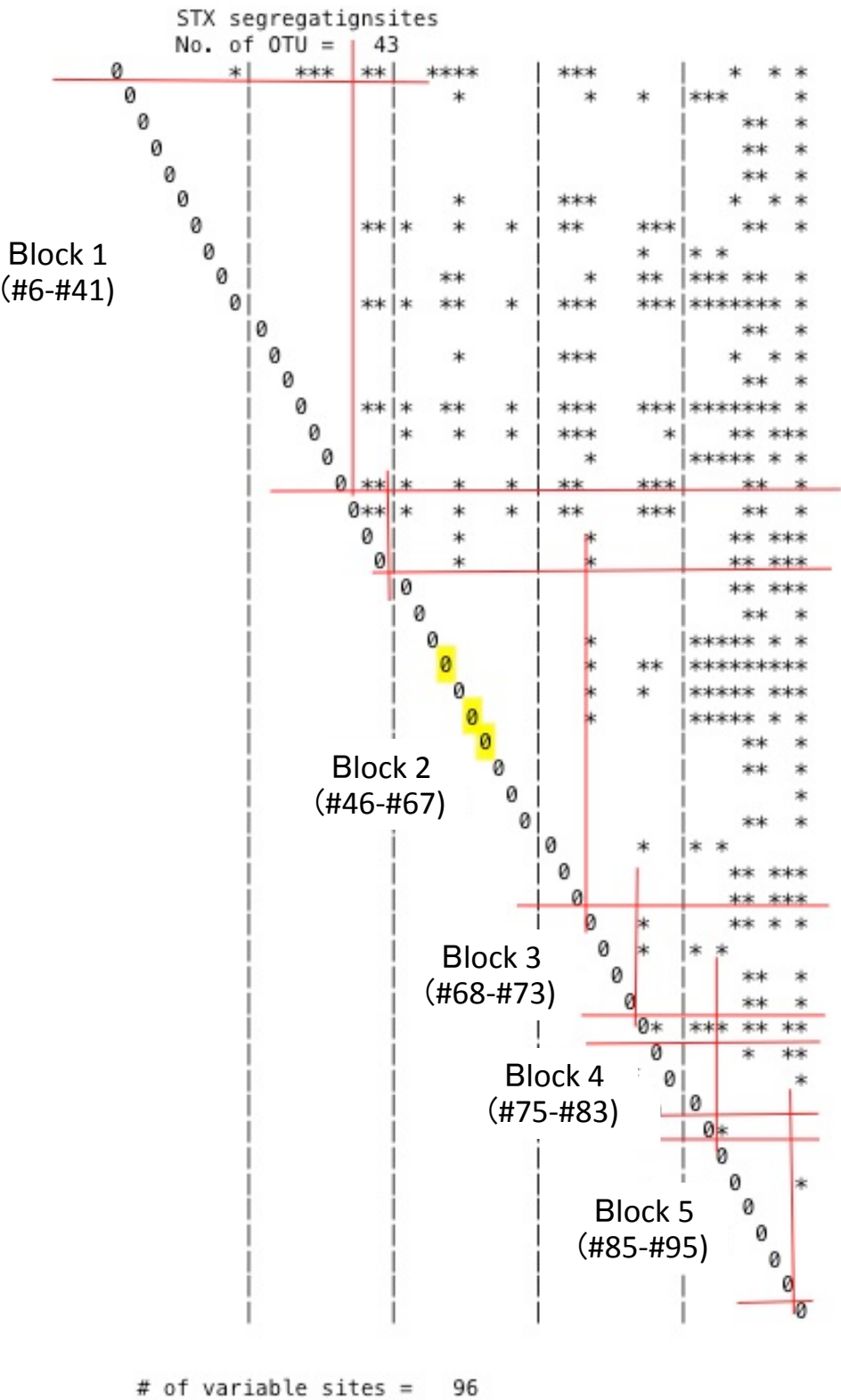

Supplement: S3 Fig — Among the 96 SNPs in D63, we placed SNP sites that are shared by more than two D63 haplotypes and are not compatible to all sites. The resulting 49 SNP sites were used for the four-gamete test. The pair of sites under a linkage break is represented by an asterisk. Five haplotype blocks were identified. The positions of the three promoter SNPs are highlighted by yellow. (PDF) [file pone.0200278.s003.pdf]

S4 Fig. ADMIXTURE analysis.

A

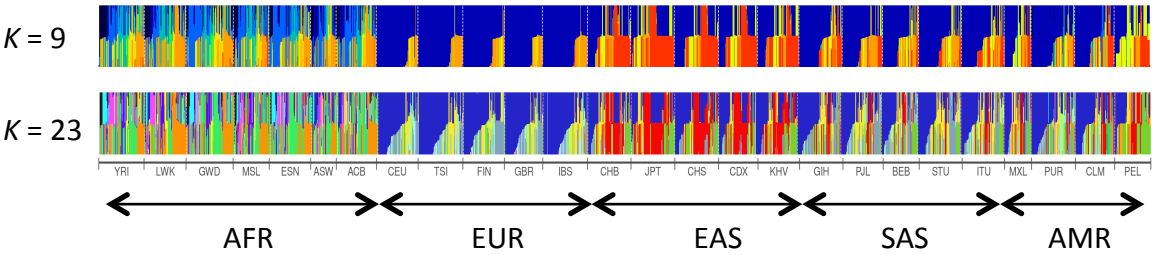

B

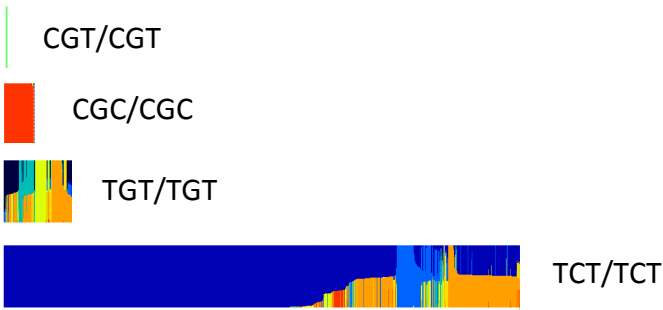

C

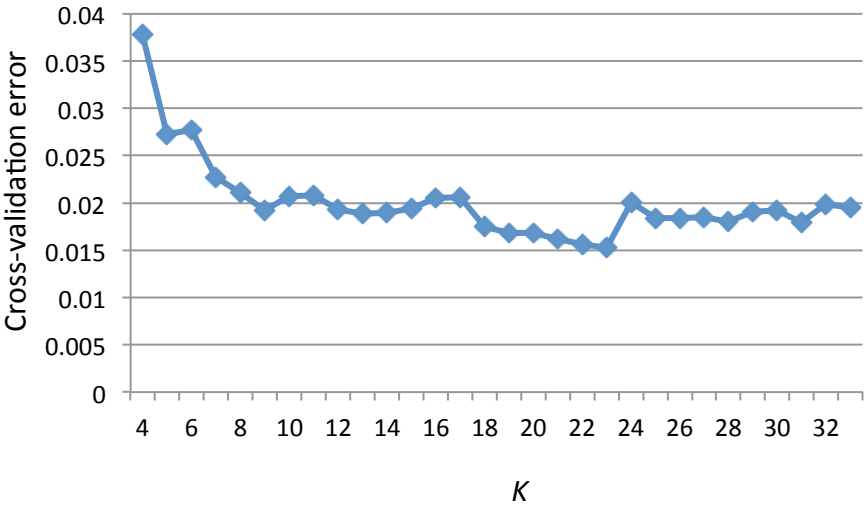

Supplement: S4 Fig — (A) ADMIXTURE pattern is not changed by increasing number of postulated ancestral populations (K). (B) ADMIXTURE pattern (K = 9) sorted by promoter types shows that the CGC type is homogeneous. (C) Cross validation error does not change with K ≥ 9. (PDF) [file pone.0200278.s004.pdf]

S5 Fig. Relative site frequency spectrum (rSFS) in the 54-kb region.

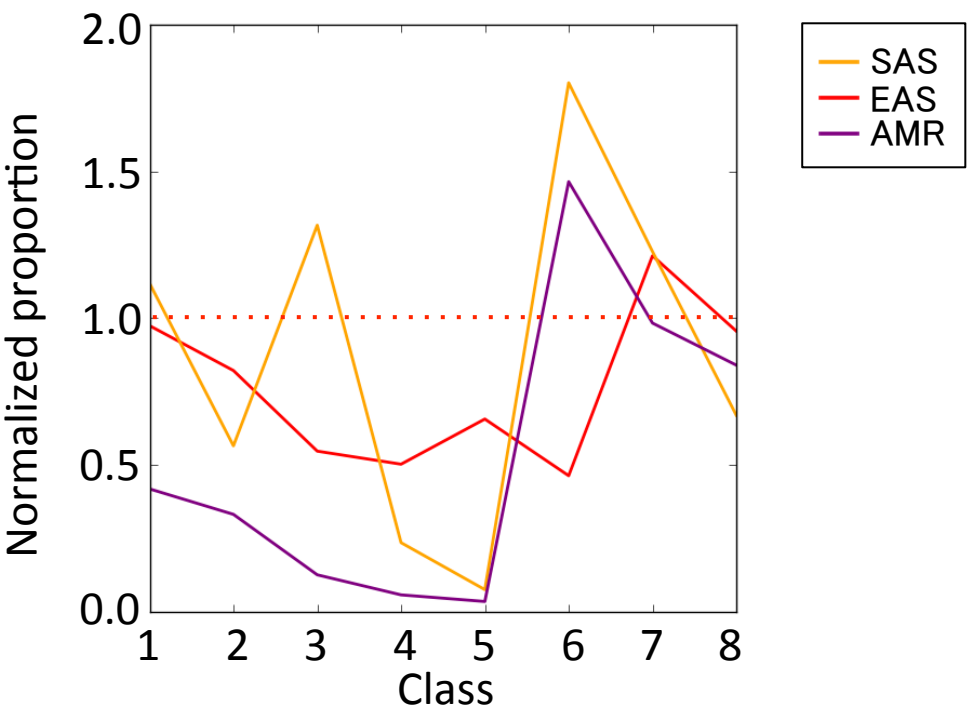

Supplement: S5 Fig — rSFS was defined as the ratio of observed-to-simulated proportions of SFS under the demographic model [17] using μ = 1.2 × 10^−8^ per site per generation. (PDF) [file pone.0200278.s005.pdf]

S6 Fig. Neighbor-joining trees for the five haplotype blocks in  $D_{63}$ .

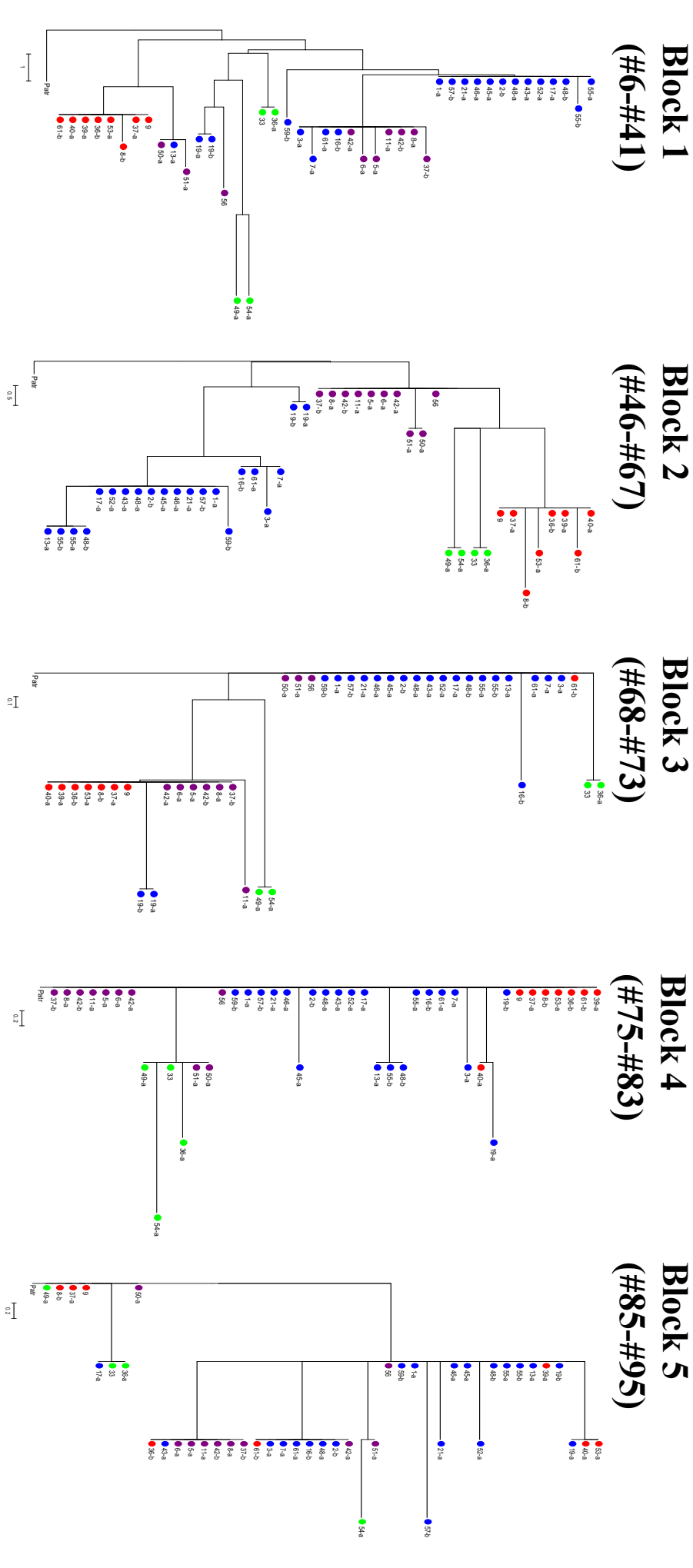

Supplement: S6 Fig — Neighbor-joining trees were constructed using the five haplotype blocks detected by the four-gamete test in D63. The sequences of each promoter type are highlighted by colors [TGT-type (purple), TCT-type (blue), CGT-type (green), and CGC -type (red)]. (PDF) [file pone.0200278.s006.pdf]

S7 Fig. Promoter type of archaic humans.

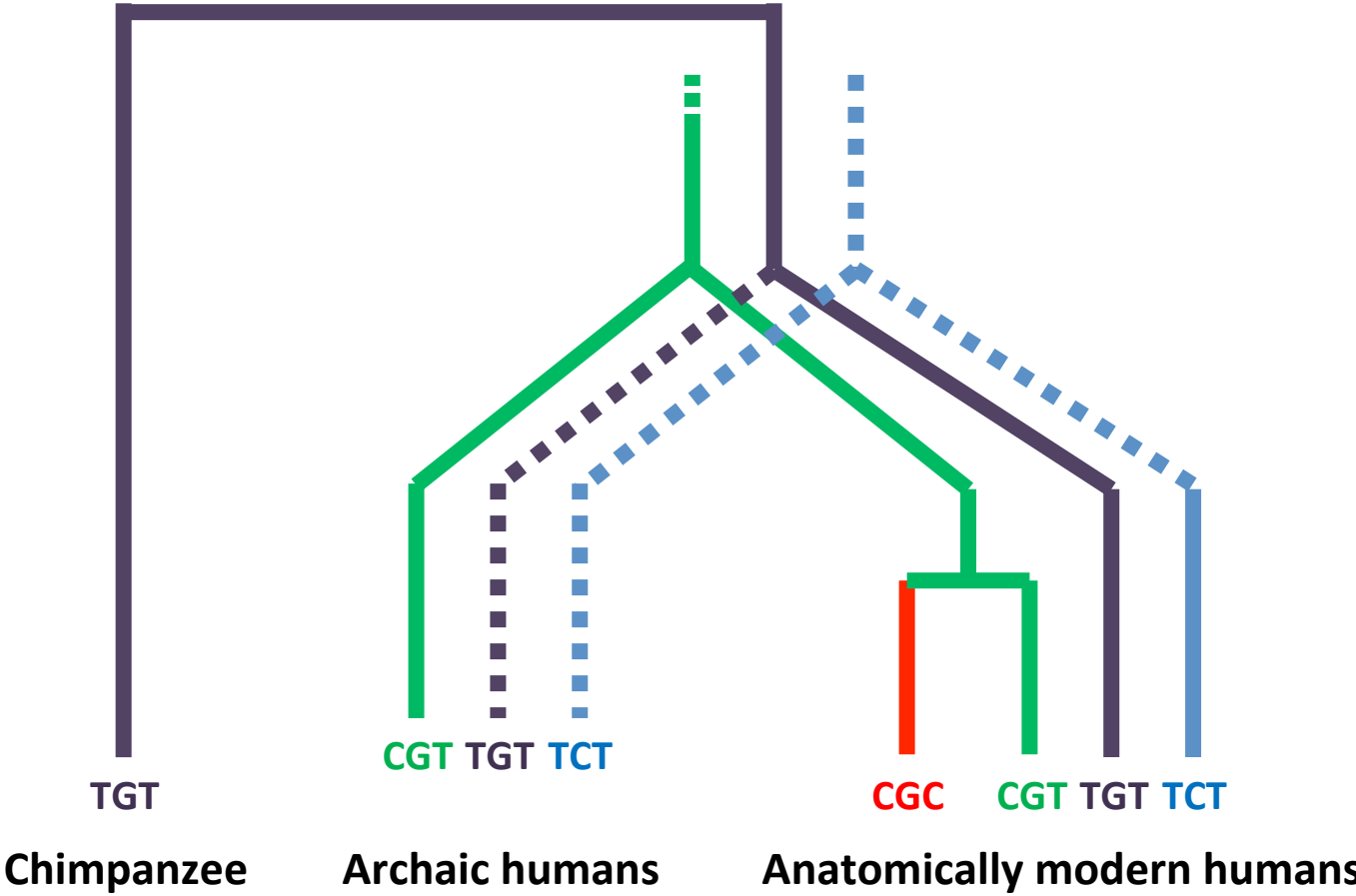

Supplement: S7 Fig — The emergence time of the CGC-type lineage is estimated to 455 thousand years ago (kya) (Fig 5), which is much later than the time of the population split of archaic humans from anatomically modern humans (AMHs) (550–765 kya; [41]). Moreover, the CGC-type has not been identified in archaic human genomes (data not shown). This indicates that the CGC-type emerged uniquely in AMHs. Recently, it has been reported that adaptive haplotypes were introduced from archaic humans to AMHs by introgression [24, 52]. However, the selective sweep by the CGC-type does not show this. Promoter type identified from a single individual known as Denisovan, an archaic human who lived in Asia, is classified as a member of the CGT-type. In addition, two Neanderthal individuals (Vindija and Altai) are homozygous for the CGT-type (data not shown), which implies that the frequency of the CGT-type in archaic humans might be considerably higher than in AMHs (1.5% in D1000). (PDF) [file pone.0200278.s007.pdf]

S8 Fig. Demographic events involved in the selective sweep by the CGC type.

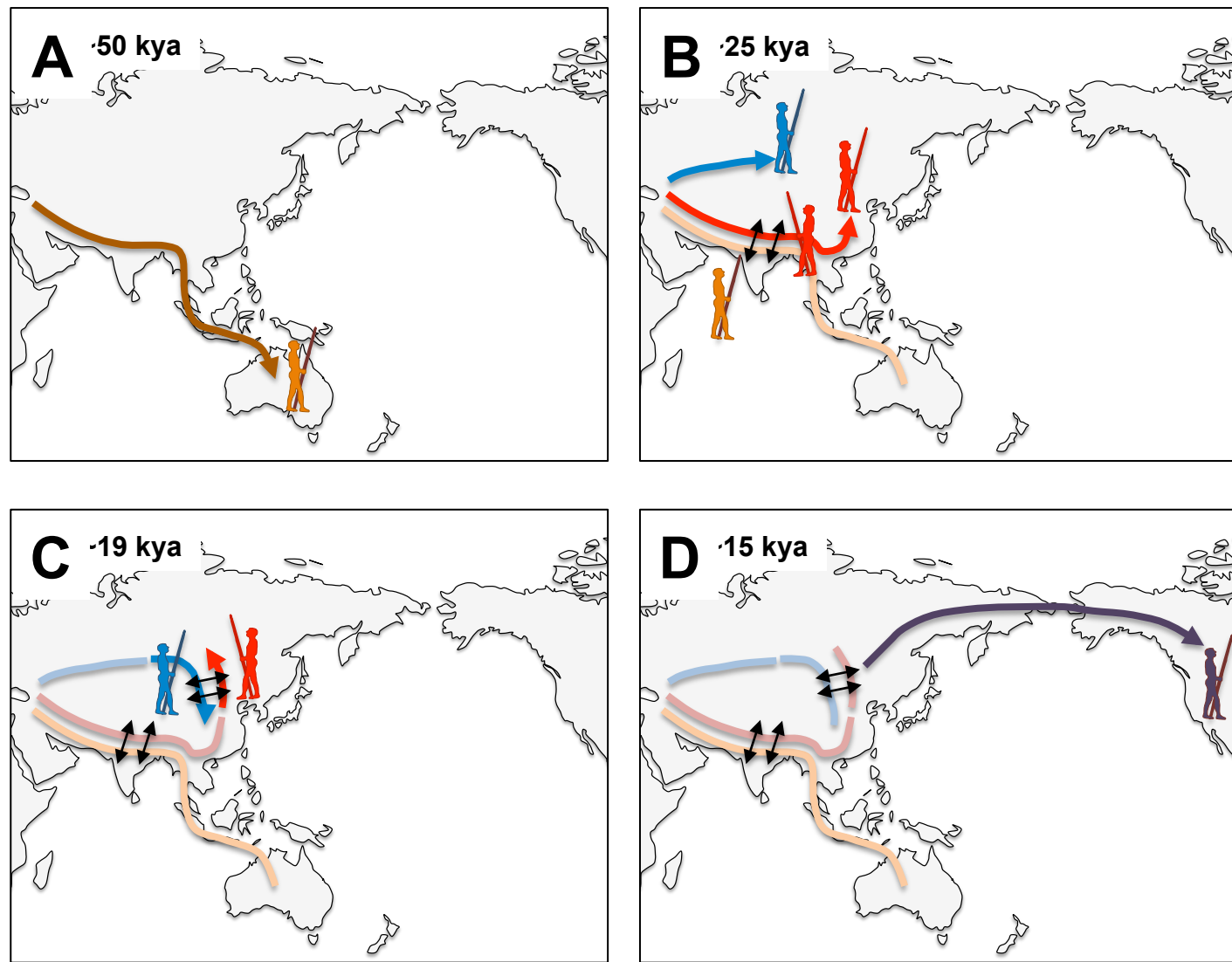

Supplement: S8 Fig — In the out-of-Africa migration, anatomically modern humans (AMHs) migrated into the Eurasian continent by three major dispersals [42, 43]. The north dispersal reached into North Asia via Central Asia by 27 thousand years ago (kya) [47], while the other two other dispersals (earlier and later south dispersals) occurred at different times in the same route passing through South Asia into Southeast Asia. (A) In the earlier south dispersal, people finally migrated into the Australian continent from Southeast Asia at least 50 kya or possibly 65 kya [53]. (B) In the later south dispersal, people finally migrated into East Asia from Southeast Asia at least 25 kya [46]. During this later south dispersal, massive admixture occurred between the people already settled in South Asia and Southeast Asia by the earlier south dispersal (earlier south migrants) and those migrated by the later south dispersal (later south migrants) [46]. The people that migrated into North Asia by the north dispersal (north migrants) and later south migrants had been unable to migrate further because of cold environments that appeared during the Last Glacial Maximum (LGM). (C) After the LGM, they started moving extensively northward and southward in the eastern part of Eurasia [54–60]. These migrations caused frequent close encounters between later south migrants and north migrants, and resulted in massive admixture in the eastern part of Eurasia, as shown by the unique genetic structure (i.e., dual genetic structure) of East Asian populations from mitochondrial genome and Y chromosome analyses [54, 61]. (D) The people who underwent admixture in the eastern part of Eurasia, simultaneously migrated into the American continent with the appearance of the Bering land bridge (Beringia) around 15 kya. This is suggested by the finding that Native Americans have mixed origins resulting from admixture between people related to East Asians and Western Eurasians [62]. Thus, present-day populations showing the s [file pone.0200278.s008.pdf]

S9 Fig. A neighbor-joining tree for all the CGC haplotype sequences.

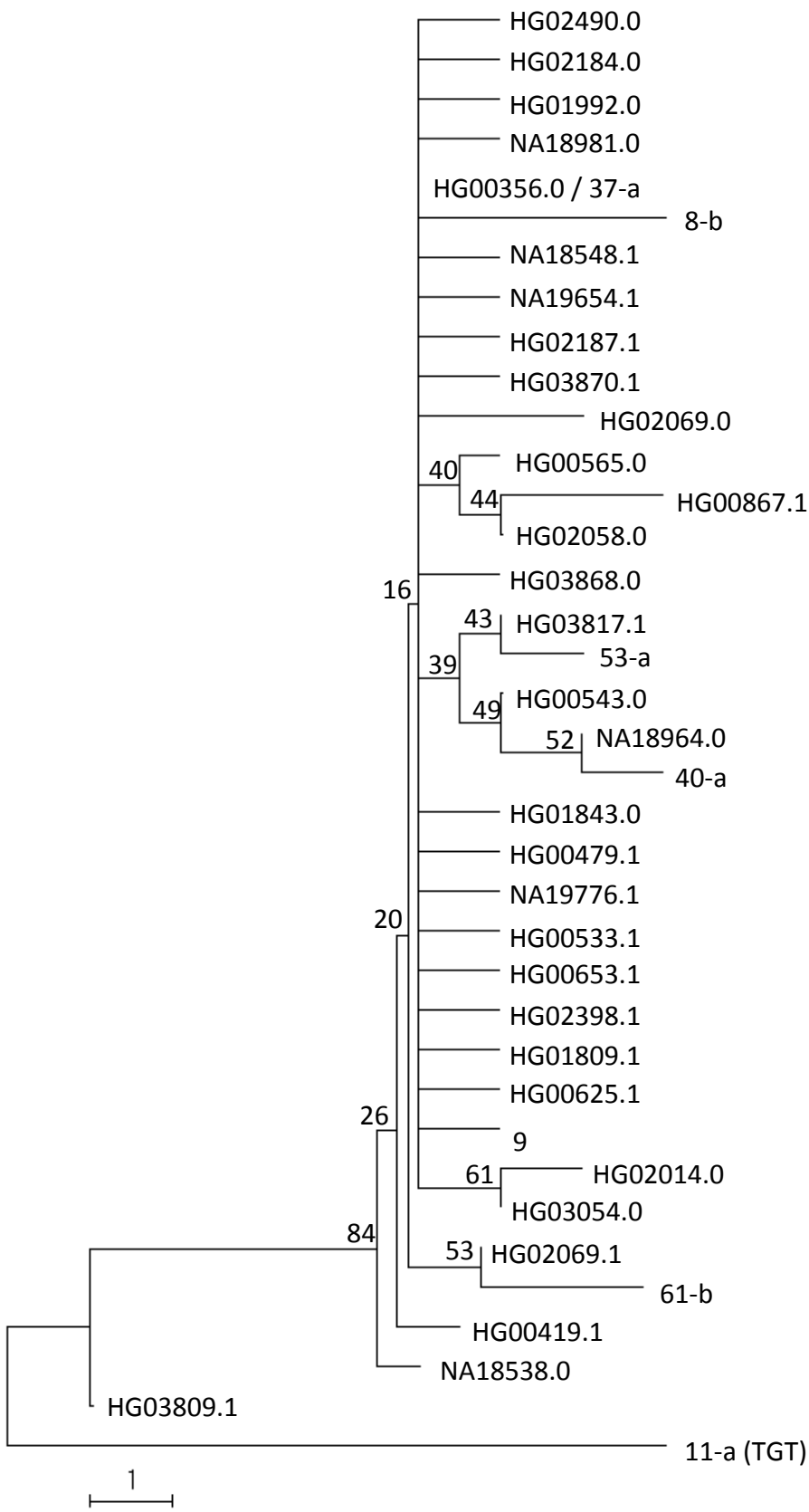

Supplement: S9 Fig — A neighbor-joining tree was constructed using the SNP data of D63 and D1000 with MEGA7 software [63]. The overlapped part (9 kb) between the 10-kb and the 18-kb regions was used (Fig 1; S7 and S8 Tables). A TGT haplotype (11-a) was used as an outgroup. Total 47 segregating sites were involved. The percentage of replicate trees in which associated taxa clustered together in the bootstrap test (1,000 replicates) is shown next to the branches. The tree is drawn to scale, with branch lengths in the same units as evolutionary distances used to infer the phylogenetic tree. Evolutionary distances were calculated using the number of differences method, and are in units of number of base differences per sequence. (PDF) [file pone.0200278.s009.pdf]
