# Supplementary material for: Positive selection on schizophrenia-associated ST8SIA2 gene in post-glacial Asia
Source: PLoS One. 2018 Jul 25;13(7):e0200278. doi: 10.1371/journal.pone.0200278 (PMC6059407; doi:10.1371/journal.pone.0200278)
Supplement: S1 Table — All 63 samples are listed with IDs, and the meta- and sub-populations to which they belong, repository numbers at Coriell Cell Repositories, and genotypes at the three promoter SNPs. (PDF) [file pone.0200278.s010.pdf]

S1 Table. Promoter types for the 63 human samples.

ID	Meta–population	Population	Repository No.	Genotype
1	AMR	Mayan	NA10975	TGT/TCT
2			NA10976	TCT/TCT
3			NA10978	TCT/TCT
4			NA10979	TGT/TCT
5		Karitiana	NA10965	TGT/TGT
6			NA10967	TGT/CGC
7			NA10968	TGT/TCT
8			NA10969	TGT/CGC
9		Surui	NA10970	CGC/CGC
10			NA10972	CGC/CGC
11			NA10974	TGT/CGC
12		Waorani	NA11776	TCT/TCT
13		Quechua	NA11197	TCT/TCT
14			NA11198	TGT/TCT
15			NA11199	TGT/TCT
16			NA11200	TCT/TCT
17	EUR	Adygei	NA13617	TCT/TCT
18			NA13618	TCT/TCT
19			NA13619	TCT/TCT
20			NA13620	TCT/TCT
21		Russian	NA13820	TCT/TCT
22			NA13838	TGT/TCT
23			NA13849	TCT/TCT
24			NA13877	TCT/CGC
25	MDE	Druze	NA11521	TCT/TCT
26			NA11522	TCT/TCT
27			NA11523	TCT/TCT
28			NA11524	TCT/TCT
29	EAS	Ami	NA13607	TCT/CGC
30			NA13608	TCT/TCT
31			NA13609	TCT/TCT
32			NA13610	TCT/CGT
33		Atayal	NA13597	CGT/CGT
34			NA13598	CGC/CGC
35			NA13599	TCT/CGC
36			NA13600	CGT/CGC
37		Southern Chinese	NA11322	TGT/CGC
38			NA11324	TGT/TCT
39		Japanese	NA11587	TCT/CGC
40			NA11589	TCT/CGC
41			NA11590	CGC/CGC
42		Khmer Cambodian	NA11373	TGT/TGT
43			NA11376	TCT/CGC
44	MLN	Melanesian	NA10539	TGT/TGT
45			NA10540	TGT/TCT
46			NA10541	TCT/CGT
47			NA10542	TCT/TCT
48	AFR	Biaka	NA10470	TCT/TCT
49			NA10472	TGT/CGT
50			NA10473	TGT/TGT
51		Mbuti	NA10493	TGT/TGT
52			NA10494	TGT/TCT
53			NA10495	TGT/CGC
54		Yoruba	NA18523	TGT/CGT
55			NA18853	TCT/TCT
56			NA19208	TGT/TGT
57	EUR	CEPH/ UTAH	NA10831	TCT/TCT
58			NA11831	TGT/TCT
59			NA06985	TCT/TCT
60	EAS	Han Chinese from Beijing	NA18532	TGT/TCT
61			NA18611	TCT/CGC
62			NA18552	TCT/CGC
63			NA18526	TGT/TGT
