# Supplementary material for: Positive selection on schizophrenia-associated ST8SIA2 gene in post-glacial Asia
Source: PLoS One. 2018 Jul 25;13(7):e0200278. doi: 10.1371/journal.pone.0200278 (PMC6059407; doi:10.1371/journal.pone.0200278)
Supplement: S2 Table — The sequences of primers used in this study are listed. (PDF) [file pone.0200278.s011.pdf]

S2 Table. Primer sequences.

Primer names	Nucleotide sequences
STXF1	5’-ACATTCCATGTAGCTTCTCCAACAG-3’
STXR2	5'-TAGGAGGGGAGGTGGGGGAAG-3'
STXF1H	5’-GAAAATCCAAGGCAGGAAAAGAGAC-3’
STXR1H	5’-GTACAGACTTTCCCGAAATGTCTC-3’
STXF2H	5’-CTTCCTCATCTTCGCAGACATCTC-3’
STXR2H	5’-TATTTATCCAACAGATGTTTATTGAATGC-3’
STXF3H	5’-GCTCTGTAAAAGGATGGCTGTTTC-3’
STXR3H	5’-ATAGCCAGACTGTGCTCTTCACTG-3’
STXF0H-2	5’-CTGAAGATGGGAGAGAAAAATCCTAT-3’
STXR0H-2	5’-TATTTATCCAACAGATGTTTATTGAATGC-3’
STXF8	5'-CCCTTGACGCTGTGCTGCTTC-3'
STXR6	5'-GCGTGTGCCTGTCTGTGTGTG-3'
STXproF1E	5'-GTACGCTATGACGAATTCAGGACATTCCATGTAGCTTCTCCAA-3'
STXproR1B	5'-ATCGTAGCTAGCGGATCCCCGATTTCTTCTTCGATCTCTGA-3'
pmST8SIA2-noTM	5’-GGTGGGTTCGCGCCCGCCGGGACCCA-3’
MetLuc2-ATG	5’-ATGGACATCAAGGTGGTGTTCACCTG-3’
