# Supplementary material for: Positive selection on schizophrenia-associated ST8SIA2 gene in post-glacial Asia
Source: PLoS One. 2018 Jul 25;13(7):e0200278. doi: 10.1371/journal.pone.0200278 (PMC6059407; doi:10.1371/journal.pone.0200278)
Supplement: S3 Table — (PDF) [file pone.0200278.s012.pdf]

S3 Table. Promoter types frequencies in the D_{1000} sequence dataset.

Meta-population	Sub-population	Population name	CGC	TGT	TCT	CGT	Others	Sum
AFR	ACB	African Caribbeans in Barbados	2	80	103	7	0	192
	ASW	Americans of African Ancestry in SW USA	2	52	64	2	2	122
	ESN	Esan in Nigeria	0	83	109	3	3	198
	GWD	Gambian in Western Divisions in the Gambia	1	100	109	8	8	226
	LWK	Luhya in Webuye, Kenya	2	115	71	8	2	198
	MSL	Mende in Sierra Leone	4	86	70	5	5	170
	YRI	Yoruba in Ibadan, Nigeria	0	96	99	13	8	216
EUR	CEU	Utah Residents (CEPH) with Northern and Western European Ancestry	0	8	190	0	0	198
	FIN	Finnish in Finland	1	9	188	0	0	198
	GBR	British in England and Scotland	0	6	176	0	0	182
	IBS	Iberian Population in Spain	0	5	207	1	1	214
	TSI	Toscani in Italia	1	5	208	0	0	214
EAS	CHB	Han Chinese in Beijing, China	60	19	125	2	0	206
	CHS	Southern Han Chinese	67	26	114	1	2	210
	CDX	Chinese Dai in Xishuangbanna, China	68	26	89	2	1	186
	JPT	Japanese in Tokyo, Japan	91	31	84	2	0	208
	KHV	Kinh in Ho Chi Minh City, Vietnam	63	31	103	1	0	198
SAS	BEB	Bengali from Bangladesh	13	26	131	2	0	172
	GIH	Gujarati Indian from Houston, Texas	21	20	160	5	0	206
	ITU	Indian Telugu from the UK	19	24	160	1	0	204
	PJL	Punjabi from Lahore, Pakistan	14	17	159	2	0	192
	STU	Sri Lankan Tamil from the UK	9	22	172	1	0	204
AMR	CLM	Colombians from Medellin, Colombia	25	23	136	4	0	188
	MXL	Mexican Ancestry from Los Angeles USA	16	23	88	1	0	128
	PEL	Peruvians from Lima, Peru	32	50	88	0	0	170
	PUR	Puerto Ricans from Puerto Rico	9	30	166	3	0	208
Sum			520	1013	3369	74	32	5008
