# Supplementary material for: Positive selection on schizophrenia-associated ST8SIA2 gene in post-glacial Asia
Source: PLoS One. 2018 Jul 25;13(7):e0200278. doi: 10.1371/journal.pone.0200278 (PMC6059407; doi:10.1371/journal.pone.0200278)
Supplement: S4 Table — FST values were calculated for SNPs in the 54-kb region between meta-populations. Mean (mean FST), standard deviation (Std of FST), and maximum values (maximum) of FST are shown with the number of segregating sites (S) and 90th and 95th percentiles of FST. FST values for the three promoter SNPs (SNP1–3) were also calculated. FST values highlighted in salmon pink are greater than the 95th percentile, while those in orange are greater than the 90th percentile. (PDF) [file pone.0200278.s013.pdf]

S4 Table. *Fst* values for SNPs in the *ST8SIA2* promoter region.

	EUR_AFR	EAS_EUR	EAS_AFR	AMR_AFR	SAS_AFR	EUR_AMR	EUR_SAS	EAS_AMR	EAS_SAS	AMR_SAS
S	1097	782	1178	1089	1171	770	745	873	772	848
Mean Fst	0.030	0.031	0.024	0.020	0.023	0.015	0.010	0.009	0.016	0.005
Std of fst	0.073	0.082	0.059	0.048	0.056	0.037	0.025	0.023	0.041	0.012
Maximum	0.470	0.415	0.466	0.380	0.467	0.254	0.142	0.166	0.317	0.139
95%	0.200	0.270	0.160	0.120	0.140	0.640	0.620	0.160	0.120	0.020
90%	0.090	0.080	0.070	0.060	0.060	0.130	0.070	0.050	0.090	0.010
SNP1	0.034	0.349	0.265	0.045	0.016	0.120	0.080	0.129	0.186	0.007
SNP2	0.458	0.415	0.002	0.090	0.206	0.231	0.119	0.061	0.166	0.031
SNP3	0.021	0.341	0.284	0.055	0.022	0.109	0.069	0.137	0.196	0.008
