# Supplementary material for: Positive selection on schizophrenia-associated ST8SIA2 gene in post-glacial Asia
Source: PLoS One. 2018 Jul 25;13(7):e0200278. doi: 10.1371/journal.pone.0200278 (PMC6059407; doi:10.1371/journal.pone.0200278)
Supplement: S5 Table — (A) Genetic variability in three regions in D1000. The 54-kb region was separated into regions A, B and C of each 18 kb length. Region B corresponds to the 18-kb region (Fig 1) that spans the three SNPs and sandwiched between weak recombination hotspots. (B) Genetic variability in D63. Note: aThe expected haplotype (allele) number in a sample of n chromosomes with estimated θw and θ values under the assumption of no recombination within each region [64]. In an equilibrium population of effective size Ne, both θw and θ reflect scaled-per-site neutral mutation rate (4Neμ) estimated from the number of segregating sites (Sn) or nucleotide diversity (π). bTajima’s D and cFay and Wu’s H were calculated using Dnasp. Statistical significance was also assessed based on 1,000 simulations using Dnasp under free recombination (for D1000) or mean recombination rate in this region (for D63) calculated by LDhat (ρ = 4Ner = 13.2, where Ne represents effective population size and r recombination rate per gene). Tests marked with asterisks were significant (P < 0.05). dSquared correlation coefficient is approximated as r2 = D2/(pAqApBqB), where pA and pB are allele frequencies at sites A and B, respectively, and pA + qA = pB + qB = 1. The average squared correlation coefficient was calculated from all pairs of polymorphic sites within a region in each population. The scaled recombination rate, ρ [65, 66], was calculated from r2 using E(r2)≈σd2≈10+ρ22+13ρ+ρ2≈10+ρ(11+ρ)(2+ρ)≈12+ρ. Values of χ2 = kr2 were no smaller than 10 [with a minimum value of 13.2 in Region C of Africa (AFR)], implying significant linkage disequilibrium (P < 0.0003) in each population despite Hn >> E(Hn). *P < 0.05. (PDF) [file pone.0200278.s014.pdf]

S5 Table. Genetic variability in the *ST8SIA2* locus.

## (A) Genetic variability in three regions in $D_{1000}$.

| Region A (18000 bp) | AFR ($n$ = 1322) | EUR ($n$ = 1006) | SAS ($n$ = 978) | EAS ($n$ = 1008) | AMR ($n$ = 694) |
|---|---|---|---|---|---|
| $H_n$ | 389 | 207 | 260 | 214 | 198 |
| $E(H_n)$[a] | (136, 100) | (84, 68) | (88, 73) | (84, 84) | (89, 76) |
| $S_n$ ($\theta_w$) | 300 (0.21%) | 167 (0.12%) | 170 (0.13%) | 160 (0.12%) | 191 (0.15%) |
| $\pi$ | 0.14% | 0.09% | 0.10% | 0.12% | 0.12% |
| D[b] | -0.987 | -0.721 | -0.574 | 0.103 | -0.628 |
| H[c] | 6.297 | -7.041 | -4.456 | 4.580 | 1.351 |
| $r^2$ ($\rho$)[d] | 0.014 (69.4) | 0.034 (27.0) | 0.032 (29.4) | 0.041 (22.3) | 0.030 (31.7) |
| Region B (17981 bp) | | | | | |
| $H_n$ | 257 | 160 | 184 | 142 | 144 |
| $E(H_n)$ | (136, 95) | (89, 43) | (88, 67) | (84, 73) | (97, 67) |
| $S_n$ ($\theta_w$) | 292 (0.21%) | 178 (0.13%) | 175 (0.13%) | 160 (0.12%) | 217 (0.17%) |
| $\pi$ | 0.13% | 0.05% | 0.09% | 0.10% | 0.10% |
| D | -1.088 | -1.842* | -0.983 | -0.372 | -1.281 |
| H | 4.071 | -17.04* | -1.157 | -2.673 | -4.725 |
| $r^2$ ($\rho$) | 0.021 (45.1) | 0.040 (22.8) | 0.032 (28.9) | 0.040 (22.7) | 0.039 (23.3) |
| Region C (18000 bp) | | | | | |
| $H_n$ | 346 | 173 | 239 | 241 | 188 |
| $E(H_n)$ | (145, 101) | (84, 79) | (88, 88) | (94, 89) | (89, 85) |
| $S_n$ ($\theta_w$) | 318 (0.23%) | 159 (0.12%) | 168 (0.13%) | 184 (0.14%) | 193 (0.15%) |
| $\pi$ | 0.14% | 0.11% | 0.13% | 0.13% | 0.14% |
| D | -1.116 | -0.093 | 0.065 | -0.079 | -0.261 |
| H | -10.39 | -22.13 | -5.913 | -6.361 | -9.227 |
| $r^2$ ($\rho$) | 0.010 (96.7) | 0.047 (19.1) | 0.032 (29.0) | 0.030 (31.2) | 0.029 (32.8) |

| The 54-kb region (53981 bp) | AFR ($n$ = 1322) | EUR ($n$ = 1006) | SAS ($n$ = 978) | EAS ($n$ = 1008) | AMR ($n$ = 694) |
|---|---|---|---|---|---|
| $H_n$ | 769 | 576 | 679 | 609 | 435 |
| $E(H_n)$ | (297, 221) | (182, 138) | (190, 160) | (182, 182) | (191, 160) |
| $S_n$ ($\theta_w$) | 910 (0.22%) | 504 (0.12%) | 513(0.13%) | 504 (0.12%) | 601 (0.16%) |
| $\pi$ | 0.14% | 0.08% | 0.10% | 0.12% | 0.12% |
| D | -1.079 | -0.941 | -0.515 | -0.117 | -0.760 |
| H | 14.01 | -30.79 | -3.64 | 4.51 | 0.97 |
| $r^2$($\rho$) | 0.009 (109.9) | 0.019 (50.1) | 0.015 (64.8) | 0.020 (48.8) | 0.018 (54.7) |

S5 Table. Genetic variability in the *ST8SIA2* locus.

## (B) Genetic variability in $D_{63}$.

| The 10-kb region (10111 bp) | AFR ($n$ = 12) | EUR ($n$ = 13) | MDE ($n$ = 7) | EAS ($n$ = 34) | AMR ($n$ = 25) |
|---|---|---|---|---|---|
| $H_n$ | 11 | 9 | 2 | 18 | 14 |
| $E(H_n)^a$ | (9, 9) | (9, 8) | (3, 3) | (15, 16) | (13, 13) |
| $S_n$ ($\theta_w$) | 55 (0.18%) | 35 (0.11%) | 2 (0.01%) | 40 (0.10%) | 37 (0.10%) |
| $\pi$ | 0.17% | 0.09% | 0.01% | 0.11% | 0.11% |
| $D^b$ | -0.398 | -1.003 | 0.687 | 0.540 | 0.521 |
| $H^c$ | 6.364 | -7.462 | -1.429* | -0.670 | -1.997 |
| $r^2$ $(\rho)^d$ | 0.188(3.25) | 0.240 (2.16) | 1.000 (-1.0) | 0.176 (3.67) | 0.196 (3.10) |
