# Supplementary material for: Positive selection on schizophrenia-associated ST8SIA2 gene in post-glacial Asia
Source: PLoS One. 2018 Jul 25;13(7):e0200278. doi: 10.1371/journal.pone.0200278 (PMC6059407; doi:10.1371/journal.pone.0200278)
Supplement: S6 Table — HTLs for CC and TT homozygotes are shown together with the number of tracts (number of homozygous individuals). Total HTL and HTL in the left-side and right-side of cores were measured separately. HTL was also calculated by pairwise comparison using all chromosomes within a meta-population. (PDF) [file pone.0200278.s015.pdf]

S6 Table. Homozygosity tract length (HTL) in meta-populations.

HTLs of CC and TT homozygotes

Population	Number of CC	Mean HTL of CC (bp) (STD)			Number of TT	Mean HTL of TT (bp) (STD)		
		Total	Left	Right		Total	Left	Right
EAS	60	25517 (15755)	16057 (11764)	9459 (9484)	211	11415 (14438)	6069 (7655)	5346 (9547)
SAS	5	37497 (29468)	20203 (6852)	17294 (25813)	410	9534 (12135)	5540 (6705)	3994 (7948)
AMR	6	29859 (21495)	14921 (6700)	14938 (20206)	267	8082 (12057)	4782 (7249)	3300 (7658)

HTLs from the pairwise comparison using all chromosomes

EAS	61105	28224 (16500)	17981 (11706)	10243 (11082)	214649	11594 (15231)	6379 (8659)	5215 (9870)
SAS	2798	26631 (16574)	16604 (10257)	10027 (12555)	405631	9108 (10776)	5618 (6626)	3490 (6478)
AMR	3056	35491 (20163)	19413 (13385)	16078 (14192)	186002	7726 (12233)	4338 (6863)	3388 (7611)
