# Supplementary material for: Positive selection on schizophrenia-associated ST8SIA2 gene in post-glacial Asia
Source: PLoS One. 2018 Jul 25;13(7):e0200278. doi: 10.1371/journal.pone.0200278 (PMC6059407; doi:10.1371/journal.pone.0200278)
Supplement: S9 Table — Distribution of the 31 CGC haplotypes in D1000 are shown. The haplotypes are defined using the overlapped part (9 kb) between the 10-kb and the 18-kb regions. (PDF) [file pone.0200278.s018.pdf]

S9 Table. Distributions of the CGC type in D_{1000}.

	AFR							EUR					EAS					SAS					AMR				SUM
Halplotype name	ACB	ASW	ESN	GWD	LWK	MSL	YRI	CEU	FIN	GBR	IBS	TSI	CHB	CHS	CDX	JPT	KHV	BEB	GIH	ITU	PJL	STU	CLM	MXL	PEL	PUR	
NA18964.0																1											1
HG03817.1																		1									1
HG00356.0	1	2			2	1			1				51	55	59	85	54	10	20	16	12	9	25	14	31	7	455
HG00565.0														1			2										3
HG00419.1													2	6	1	2	2									2	15
HG02069.0													1			1	1		1								4
NA18538.0													1														1
HG00543.0														1			1										2
HG02069.1																	1										1
HG03809.1																		1									1
HG01809.1															2												2
HG00533.1														1													1
HG02187.1													1		1												2
HG00653.1														1													1
NA19654.1												1												1			2
HG00625.1														1													1
NA18548.1													1														1
NA18981.0																1											1
HG02058.0																	1										1
HG02184.0															1												1
HG00867.1															1												1
HG00479.1														1	1												2
HG01843.0													1		1	1	1										4
HG03054.0						2																					2
HG03870.1																		1		1							2
HG01992.0																									1		1
HG02014.0	1			1		1																					3
HG02490.0																				1	2						3
NA19776.1																								1			1
HG02398.1													2		1												3
HG03868.0																				1							1
SUM	2	2	0	1	2	4	0	0	1	0	0	1	60	67	68	91	63	13	21	19	14	9	25	16	32	9	520
