# Supplementary material for: Positive selection on schizophrenia-associated ST8SIA2 gene in post-glacial Asia
Source: PLoS One. 2018 Jul 25;13(7):e0200278. doi: 10.1371/journal.pone.0200278 (PMC6059407; doi:10.1371/journal.pone.0200278)
Supplement: S7 Table — Segregating sites were compared with nucleotides at corresponding sites of primates [Chimpanzee (Patr) and Gorilla (Gogo)]. Dots refer to nucleotides that are identical with the chimpanzee sequence. The three promoter SNPs (SNP1–3) are highlighted in yellow. The 21 SNPs not found in D1000 (newly discovered SNPs) are highlighted in pink. The haplotypes used for the promoter assay are marked with blue. The locations of the overlapped region with the 18-kb region, Block 1–5, and the region used for the promoter assay are shown on top of the table. (PDF) [file pone.0200278.s016.pdf]

S7 Table. Alignment of 96 segregating sites in 91 haplotype sequences of D_{63}.

Overlapped region with the 18-kb region; Block 1; Block 2; Block 3; Block 4; Block 5; Promoter assay

Promoter type	Haplotype name	Sites 1–10	Sites 11–20	Sites 21–30	Sites 31–40	Sites 41–50	Sites 51–60	Sites 61–70	Sites 71–80	Sites 81–90	Sites 91–96	Number of chr
	Patr	TCTCGGCGAT	CGCCAGATAG	TATGATGTCG	AGATTCCTTT	GACACAATTC	GTTGCGCGCG	-GGGCCCCGG	GGCATTGCGC	CGGCGGAACG	AGACCT	
	Gogo	....	.C........	....	....	....	....	-.........	....	..T.N.....	......	
TGT	37-b	....T.....	.T........	..C...A...	..C....C..	A..TAC....	....	T......G..	...G......	...A......	G.G...	1
TGT	8-a	....T.....	.T........	..C...A...	..C....C..	...TAC....	....	T......G..	...G......	...A......	G.G...	8
TGT	42-b	....T.....	.T........	..C...A...	..C....C..	..TTAC....	....	T......G..	...G......	...A......	G.G...	1
TGT	11-a	....T.....	.T........	..C...A...	..C....C..	...TAC....	....	T......G.A	...G......	...A......	G.G...	1
TGT	5-a	....T.....	.T.....C..	..C...A...	..C....C..	...TAC....	....	T......G..	...G......	...A......	G.G...	1
TGT	6-a	....T.....	.T........	..C...A...	..C....CC.	...TAC....	....	T......G..	...G......	...A......	G.G...	1
TGT	42-a	....T.....	.T........	..C...A...	..C....C..	...TAC....	....	T......G..	...G......	...A.....A	G....C	1
TGT	50-a	....	.C..C.....	C.C......A	....	.G...C....	..C.......	T.........	.....C....	...A......	......	1
TGT	51-a	....	.C..C.....	C.C......A	....C.....	.G...C....	..C.......	T.........	.....C....	...A......	GT...C	1
TGT	56	.....A....	GC....G...	..C.......	.C...T.C..	.....C....	....	T.........	...G......	...A......	G.....	2
TCT	59-b	....	.CG.......	....	..C....C..	...TA.....	C....A....	T..TT.....	...G......	...A......	G.....	1
TCT	1-a	....	.C........	..C.......	..C....C..	...TA.....	C.........	T..TT.....	...G......	...A......	G.....	9
TCT	57-b	....	.C........	..C.......	..C....C..	...TA.....	C.........	T..TT.....	...G......	...A.CT...	G.....	1
TCT	21-a	....	.C........	..C.......	..C....C..	...TA.....	C.........	T..TT.....	...G......	...A...G..	G.....	1
TCT	46-a	..C.......	.C........	..C.......	..C....C..	...TA.....	C.........	T..TT.....	...G......	...A......	G.....	1
TCT	45-a	....	.C........	..C.......	..C....C..	...TA.....	C.........	T..TT.....	...G......	..TA......	G.....	3
TCT	2-b	....	.C........	..C.......	..C....C..	...TA.....	C.........	T..TT.....	...G......	...A.....A	G....C	14
TCT	48-a	....	.C........	..C.......	..C....C..	...TA.....	C.........	T..TT.....	...G......	...A.....A	G.....	2
TCT	43-a	....	.C........	..C.......	..C....C..	...TA.....	C.........	T..TT.....	...G......	...A......	G.G...	1
TCT	52-a	....	.C........	..C.......	..C....C..	...TA.....	C.........	T..TT.....	...G......	...AT.....	G.....	1
TCT	17-a	....	.C........	..C.......	..C....C..	...TA.....	C.........	T..TT.....	...G......	...A......	...T..	2
TCT	48-b	....	.C........	..C.......	..C....C..	...TA.....	C.....T...	T..TT.....	...G...T..	...A......	G.....	1
TCT	55-a	....	.C........	..C.......	..C....C..	...TA.....	C.....T...	T..TT.....	...G......	...A......	G.....	1
TCT	55-b	....	.C......G.	..C.......	..C....C..	...TA.....	C.....T...	T..TT.....	...G...T..	...A......	G....C	1
TCT	13-a	....	.C..C.....	C.C......A	....	.G.TA.....	C.....T...	T..TT.....	...G...T..	...A......	G....C	1
TCT	16-b	....T.....	.T........	..C...A...	..C....C..	...TAC....	C.........	T..TT.....	A..G......	...A.....A	G....C	1
TCT	61-a	....T.....	.T........	..C...A...	..C....C..	...TAC....	C.........	T..TT.....	...G......	...A.....A	G....C	5
TCT	7-a	....T.....	.T........	..C...A...	C.C....C..	...TAC....	C.........	T..TT.....	...G......	...A.....A	G....C	1
TCT	3-a	....T.....	.T........	..C...A...	..C....C..	...TAC....	C........C	T..TT.....	...G......	T..A.....A	G....C	1
TCT	19-a	.....A....	.C...AG...	..C.......	.......C..	.....C....	C.........	C......GC.	......A.T.	........T.	G....C	1
TCT	19-b	...A.A....	.C...AG...	..C.......	.......C..	.....C....	C.........	C......GC.	...G......	....	G....C	1
CGC	9	C.........	.C........	C.C......A	...C...C..	.....C.GC.	.C........	T......G..	...G......	...A......	......	4
CGC	37-a	....	.C........	C.C......A	...C...C..	.....C.GC.	.C........	T......G..	...G......	...A......	......	8
CGC	8-b	....	.C........	C.C.G....A	...C...C..	.....C.GC.	.C........	TAA....G..	...G......	...A......	......	1
CGC	53-a	....	.C........	C.C......A	...C...C..	.....C.GC.	.C.C......	T......G..	...G......	...A....T.	G....C	1
CGC	36-b	....	.C........	C.C......A	...C...C..	.....C.GC.	.C........	T......G..	...G......	...A......	G.G...	1
CGC	39-a	....	.C........	C.C......A	...C...C..	.....C.GC.	.C........	T......G..	...G......	...A......	G.....	1
CGC	40-a	....	.C........	C.C......A	...C...C..	.....C.GC.	.C........	T......G..	...G....T.	........T.	G....C	1
CGC	61-b	....T.....	.C........	C.C......A	...C...C..	.....C.GC.	.C........	T....T....	...G......	...A.....A	G....C	1
CGT	36-a	.T........	.C........	.GC.......	.......C.C	.....C..CA	.......C..	T.....T...	..G..C...T	...A......	...T..	1
CGT	33	....	.C........	.GC.......	.......C.C	.....C..CA	.......C..	T.....T...	..G..C....	...A......	...T..	2
CGT	54-a	......TACC	.C.T..G..C	..C..C..T.	.......C..	.....CG.C.	....T...A.	T........C	.A.GCC....	.A.A......	GT..TC	1
CGT	49-a	.......ACC	.C....G...	..CA.C.GT.	......TC..	.....CG.C.	....T...A.	T........C	.A.G.C....	...A......	......	1
