# Supplementary material for: Positive selection on schizophrenia-associated ST8SIA2 gene in post-glacial Asia
Source: PLoS One. 2018 Jul 25;13(7):e0200278. doi: 10.1371/journal.pone.0200278 (PMC6059407; doi:10.1371/journal.pone.0200278)
Supplement: S8 Table — Distribution of the 6 CGC haplotypes in D63 are shown. The haplotypes are defined using the overlapped part (9 kb) between the 10-kb and the 18-kb regions. (PDF) [file pone.0200278.s017.pdf]

S8 Table. Distributions of the CGC-type in D_{63}.

	AFR			EUR			EAS							AMR					MDE	MLN	SUM
Haplotype name	Biaka	Mbuti	Yoruba	Adygei	Russian	CEPH/ UTAH	Ami	Atayal	Southern Chinese	Han Chinese from Beijing	Japanes e	Cambodi an	Khmer	Mayan	Karitiana	Surui	Waorani	Quechua	Druze	Melanesi an	
9																4					4
37–a					1			3	1		3				1	1					10
8–b															1						1
53–a		1																			1
40–a											1										1
61–b										1											1
Total																					18
